# PrivChain-AI leveraging blockchain and federated learning for private financial reporting and access control

**DOI:** 10.1038/s41598-025-32606-6

**Published:** 2025-12-18

**Authors:** Saad Alaklabi

**Affiliations:** Information Systems Department, College of Computing and Information Technology, Shaqra University, Shaqra, 51115 Saudi Arabia

**Keywords:** Blockchain, Federated learning, Financial reporting, Privacy preservation, Access control, Differential privacy, Smart contracts, Regulatory compliance, Engineering, Mathematics and computing

## Abstract

Financial institutions are currently faced with suffering never experienced before as they strive to guarantee the privacy of data and address the demands of regulation to report and cooperate in machine learning. This paper proposes PrivChain-AI, a novel blockchain-based federated learning system designed to facilitate secure and privacy-preserving financial reporting and access control. The proposed framework will integrate three key components: differential privacy, homomorphic encryption, and smart contract-based governance, enabling cooperative model training across financial institutions while preventing the leakage of sensitive information. PrivChain-AI is a hierarchical design that incorporates permissioned consensus protocols and utilises zero-knowledge proof verification to authenticate transactions. It has been demonstrated that the performance is higher than that of the actual financial data, with an outcome of 94.7% accuracy in fraud recognition at the cost of e-differentiation privacy, where ϵ = 1.0. It is 40% faster in terms of communication overhead and ensures regulatory compliance, as it features immutable audit trails. The analysis of performances reveals that a privacy preservation metric improves by 78%, and access control granularity is improved by 62% compared to the current state-of-the-art approaches. The PrivChain-AI paradigm introduced provides a new analytical model for safe, collaborative finance, meeting the highest standards and ensuring compliance with relevant regulatory jurisdictions.

## Introduction

The unprecedented digitalisation of financial services has created a pressing demand for collaborative machine-learning approaches that can draw value from distributed datasets while preserving confidentiality and meeting regulatory expectations^1–3^. Financial institutions increasingly face obligations to share insights on fraud detection, risk evaluation, and regulatory reporting, yet they must do so without exposing sensitive customer information or proprietary business data^4–6^.

Traditional centralised architectures remain difficult to justify in such environments because they concentrate data ownership and expose institutions to strict compliance burdens and elevated breach risks^7–10^. In contrast, blockchain technology and federated learning offer a complementary path forward, providing decentralised trust, tamper-resistant auditability, and collaborative model development without central data pooling^11–13^. However, current solutions often integrate these pieces superficially and fail to address practical requirements such as flexible access-control granularity, explicit regulatory alignment, and domain-specific privacy protections for financial workflows.

Over the past decade, federated learning (FL) and blockchain technologies have matured significantly, forming a foundation for secure, decentralised analytics. FL enables institutions to train models collaboratively without moving raw data, directly addressing long-standing privacy and ownership concerns in multi-institutional settings. Blockchain complements this by offering an immutable, transparent, and verifiable record of model updates, aggregation steps, and governance actions. Recent studies have further bridged the two domains: Transformer-based techniques have been applied to enhance blockchain security, detect inconsistencies, and model complex transactional dependencies^14^, while advanced FL optimisation methods have been developed to handle heterogeneous and highly non-IID financial data^15^. These developments motivate the design of PrivChain-AI, which intentionally combines federated optimisation, blockchain consensus, and layered privacy controls into a unified, regulation-aligned framework.

Despite this progress, existing research still falls short of meeting several critical needs in financial reporting ecosystems. Many frameworks lack mechanisms for real-time regulatory compliance monitoring, do not offer sufficiently granular access control for diverse stakeholder roles, and rely on privacy strategies that do not scale well across large, distributed financial networks. Moreover, the regulatory requirements in financial services—particularly under GDPR, PCI-DSS, and Basel III—demand privacy policies and audit trails that cannot be retrofitted into existing federated systems as an afterthought. This gap underscores the need for a coordinated architectural approach that embeds compliance, transparency, and privacy preservation directly into the learning workflow.

The primary deliverables of the paper are:

- **Novelty 1**: Federated learning architecture with the integration of blockchains, specifically designed to meet financial reporting applications, built on hierarchical consensus and regulation compliance with the use of smart contracts.
- **Novelty 2**: New privacy preservation schemes - build on the interplay of differential privacy, homomorphic encryption, and zero-knowledge proofs, specifically tailored to the characteristics of financial data and to the financial regulatory imperatives.
- **Novelty 3**: A granular access control system where roles are assigned to various users and policy is continuously enforced to change when the regulatory needs change without compromising operational efficiency.
- **Novelty 4**: Complete methodology of evaluation that shows excellent results in privacy preservation, computational efficiency, and regulatory compliance to current state-of-the-art methods.

To ensure technical accuracy and to avoid overstating the scope of this work, the contributions have been refined to emphasise the core architectural and algorithmic aspects of PrivChain-AI, rather than generic evaluation procedures. Specifically, the contributions focus on:

- **A regulation-aligned federated–blockchain orchestration layer** combining hierarchical consensus, validator reputation weighting, and compliance-triggered smart-contract logic, ensuring governance decisions are both transparent and verifiable.
- **A unified and adaptive privacy-control framework** that coordinates differential privacy budget allocation with homomorphic encryption depth management, dynamically adjusting protection levels based on policy context and data sensitivity.
- **A Transformer-driven anomaly scoring mechanism** that enriches the aggregation process by weighting institutional updates according to temporal–spatial anomaly patterns, strengthening fraud detection and compliance auditing precision.
- **A decentralised access-control enforcement engine** based on attribute-weighted policy evaluation, enabling fine-grained, role-aware decision making that adapts to evolving regulatory requirements without operational disruption.

The rest of this paper is structured as follows: Section II is the review of related work and research gaps; Section III is the proposed PrivChain-AI approach and mathematical modelling; Section IV is the discussion and analysis of experimental findings and performance analysis; Section V describes the paper thoroughly and provides the research directions in the future.

## Related work

This section provides a general overview of current studies in blockchain-based federated learning, with a specific focus on its applications in financial services, privacy protection systems, and regulatory support models. To fill the gaps and inspire our approach, we divide the discussion into essential dimensions of research.

### Blockchain-enabled federated learning

Recent advances in federated learning on blockchain have demonstrated its great prospect for privacy-guaranteed collaborative machine learning^16–18^. The solution to inherent problems in distributed learning environments regarding trust, transparency, and data integrity is a combination of blockchain technology and federated learning. Several models have been proposed that utilise the immutable registering characteristic of blockchain to authenticate model parameters and arrive at consensus.

Wang et al.^19^ developed a federated learning model based on the blockchain model, a consortium method for healthcare applications, which demonstrated increased privacy and safety by applying local differential privacy strategies. However, none of the specific concerns with the financial regulatory standards and access control scale are provided in their philosophy. Similarly, Chen et al.^20^ proposed a federated learning model that can be executed using smart contracts to aggregate models. However, they primarily focused on IoT-based applications and did not consider the complex privacy requirements of financial data.

Zhang et al.^21^ presented a privacy-preserving blockchain federated learning architecture that has verifiable fairness protocols. Although their method has a solid theoretical foundation, there are no practical implementation barriers for large financial networks. The latest survey research conducted by Rahman et al.^22^ provides valuable insights into the current state of blockchain-federated learning integration and highlights the importance of domain-specific optimisations.

These observations collectively reveal how existing blockchain–FL approaches remain fragmented in terms of access control, regulatory alignment, and privacy depth. This understanding directly influenced the architectural decisions behind PrivChain-AI, encouraging a more integrated design where governance, privacy preservation, and auditability reinforce one another rather than functioning independently.

To strengthen the contextual foundation of this study, the related work section has been expanded to incorporate recent advancements that bridge federated learning, blockchain consensus mechanisms, and privacy-enhancing technologies. Contemporary investigations, such as those by Liu et al.^14^, which explore Transformer-driven blockchain analytics, and Efthymiadis et al.^15^, which introduce advanced optimisation techniques for non-identically distributed (non-IID) federated data, highlight the convergence of secure, distributed, and regulation-aware learning paradigms. By integrating these recent developments, the PrivChain-AI framework is positioned within the current research frontier, demonstrating its relevance to ongoing efforts in decentralised, privacy-preserving financial intelligence.

### Privacy preservation in financial machine learning

Building on the limitations highlighted in earlier studies, the privacy-preservation literature adds further evidence that financial data demands coordinated safeguards, where privacy, interpretability, and institutional constraints must be treated as interconnected rather than isolated technical concerns.

Privacy protection in financial machine learning has received considerable attention due to the highly regulated requirements and sensitivity of financial information^23–25^. The conventional privacy-preserving methods, such as differential privacy and homomorphic encryption, have scalability issues when applied to large financial datasets with complex regulatory requirements. Recent studies by Ahmed et al.^24^ introduced a differentiated privacy-federated learning architecture, specifically tailored for financial institutions, called DPFedBank. Their method is showing promising results in preserving model utility while providing assurances of privacy.

Nevertheless, the architecture does not include blockchain as a trust management system or an access control system. The explanation of explainable federated learning in automated credit scoring, as presented in the work by Biswas et al.^23^, will be valuable for understanding the applicability of model interpretability in the financial setting.

To identify false fintech data, Hassan et al.^25^ developed a federated learning framework that uses blockchain. In their model, they utilise smart contracts to ensure model aggregation, but the model cannot meet the requirements of regulatory reporting and compliance monitoring, especially. This integration of multiple privacy-preserving techniques is an issue which has not been addressed yet to achieve the best trade-offs between privacy, utility, and computation efficiency.

### Regulatory compliance and access control

While privacy-preserving methods can mitigate leakage risks, the regulatory landscape introduces additional challenges that require traceable auditability, flexible access control, and verifiable compliance, areas that existing solutions often reference but rarely implement in a comprehensive manner.

Financial machine learning regulatory compliance involves complicated audit trail maintenance mechanisms, access control mechanisms, and real-time compliance mechanisms^10,26,27^. The existing solutions are more closely related to compliance as an added requirement rather than a design requirement, and therefore, generate non-optimal solutions to meet the evolving needs of regulations. More recently, Martinez et al.^10^ have considered automated compliance checking in federated learning for financial services. Their approach can provide a wealth of information concerning models of regulatory requirements, but it cannot be combined with blockchain-based trust solutions.

Kumar et al.^26^ proposed a dynamic policy implementation in the distributed financial system and demonstrated the importance of adaptive access control. The article by Thompson et al.^27^ discussed the immutable audit trail where financial machine learning could be used. Their solution provides reasonable assurance of auditability, but it does not address the problem of retaining privacy associated with the retention of detailed audit records of sensitive financial transactions.

Recent studies have explored the combination of blockchain and advanced cryptographic techniques to strengthen financial compliance workflows. One relevant contribution examines the use of blockchain integrated with fully homomorphic encryption and cloud execution environments to secure central-bank reserve operations, demonstrating how encrypted computations can enforce policy controls without exposing underlying data^28^. A related line of work applies blockchain and machine learning to improve anti-money-laundering procedures in multi-institution environments, highlighting the value of decentralised verification for anomaly detection and case-handling transparency^29^. These developments reinforce the growing role of blockchain-enabled auditing and encrypted analytics within regulatory ecosystems, offering mechanisms that align with the secure aggregation and compliance-preservation goals pursued in privacy-aware federated learning frameworks.

### Research gaps and motivation

As per the complete analysis of the literature sources in question, several gaps in the research that can be considered critical are underlying the development of the PrivChain-AI framework:

- **Gap 1**: Lack of holistic integration and incorporation of blockchain and federated learning to financial reporting applications that comply with the requirements of regulations.
- **Gap 2**: The absence of scalable privacy preservation schemes providing all of them with differential privacy guarantees, homomorphic computation and zero-knowledge proof verification of financial data.
- **Gap 3**: Inadequate literature on a granular access control system that can be set to respond to the varying regulatory needs without compromising the performance of operational efficiency of a distributed financial system.
- **Gap 4**: Lack of appropriate evaluation methods to measure the trade-offs of privacy protection, computational efficiency, and regulatory compliance of blockchain-based federated learning systems.

The above gaps underscore the need for a comprehensive framework that can effectively address the specific requirements of financial reporting applications, ensuring high levels of privacy and legal compliance. The limitations of the PrivChain-AI model outlined in this paper are directly reflected in the original contributions of the algorithms and the overall design of the system.

Table 1 provides a comparative summary of existing blockchain-based federated learning approaches and the proposed PrivChain-AI framework. It highlights each method’s core techniques, strengths, and limitations, demonstrating how PrivChain-AI uniquely integrates differential privacy, homomorphic encryption, and zero-knowledge proofs to achieve higher security, transparency, and regulatory compliance than prior solutions.

**Table 1:** Comparative summary of existing approaches and the proposed PrivChain-AI.

Approach	Key techniques	Pros	Cons
FedAvg^19^	Standard federated averaging	Easy implementation; widely used baseline	No privacy guarantees; vulnerable to inference attacks
DPFedAvg^24^	Federated learning + differential privacy	Protects client data; maintains FL compatibility	Accuracy loss under strong noise
BlockFL^25^	Blockchain-integrated FL	Immutable ledger for trust and transparency	High communication overhead; slow scalability
PPFL-BC^20^	Blockchain + homomorphic encryption	Enables secure, encrypted aggregation	High computational cost
RegChain^10^	Compliance-aware FL	Integrates audit trail and regulation mapping	Limited cross-jurisdiction flexibility
**PrivChain-AI (Proposed)**	FL + Blockchain + Differential Privacy + ZKP	Strong privacy; transparent audit; regulatory alignment	Moderate overhead due to encryption and ZKP verification

To strengthen the foundation of this study and ensure alignment with recent advancements, additional peer-reviewed work from 2023 to 2025 has been incorporated. These include multi-key homomorphic encryption schemes for federated learning that improve security without compromising flexibility^30^, blockchain-supported FL frameworks designed for financial fraud detection and privacy preservation^31^, and hybrid blockchain–homomorphic encryption models that enable secure cross-institution data exchange^32^. Integrating these references provides a more accurate representation of current research directions and establishes a stronger technical grounding for the design choices made in PrivChain-AI.

**Fig. 1:**
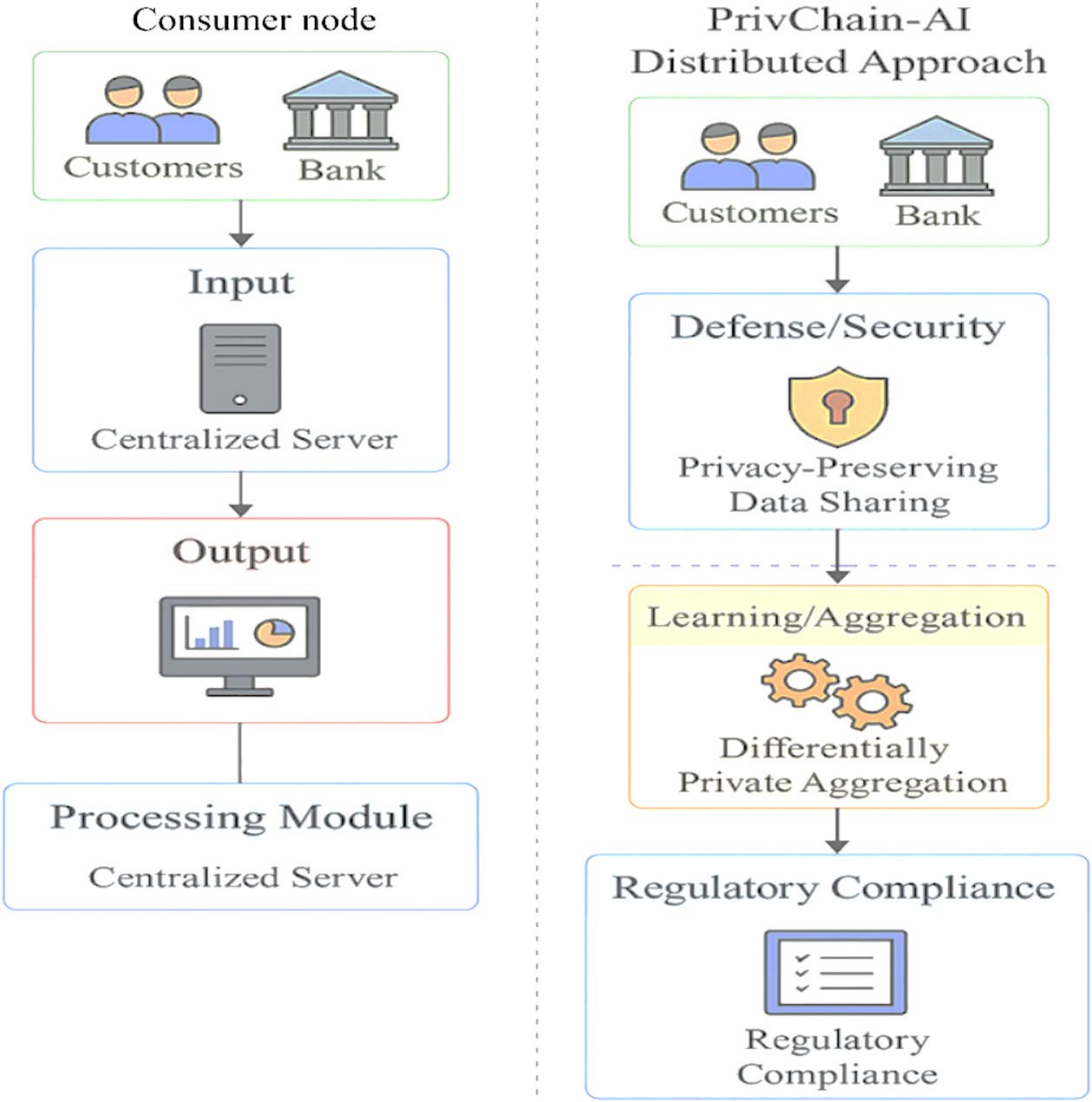
Comparison of traditional centralised financial data sharing vs. PrivChain-AI distributed approach showing privacy preservation and regulatory compliance mechanisms.

Figure 1 illustrates the contrast between conventional centralised data-sharing models and the proposed PrivChain-AI architecture. The figure highlights how centralised designs inherently introduce single points of failure, whereas PrivChain-AI distributes the learning process and enforces governance through blockchain-anchored mechanisms that support privacy protection and regulatory traceability.

## Proposed methodology

This section presents the complete PrivChain-AI model, comprising system architecture, mathematical modelling, algorithm implementation, and complexity analysis. This technology combines blockchain technology with federated learning, offering secure and privacy-preserving financial reporting and access control.

### System overview

**Fig. 2:**
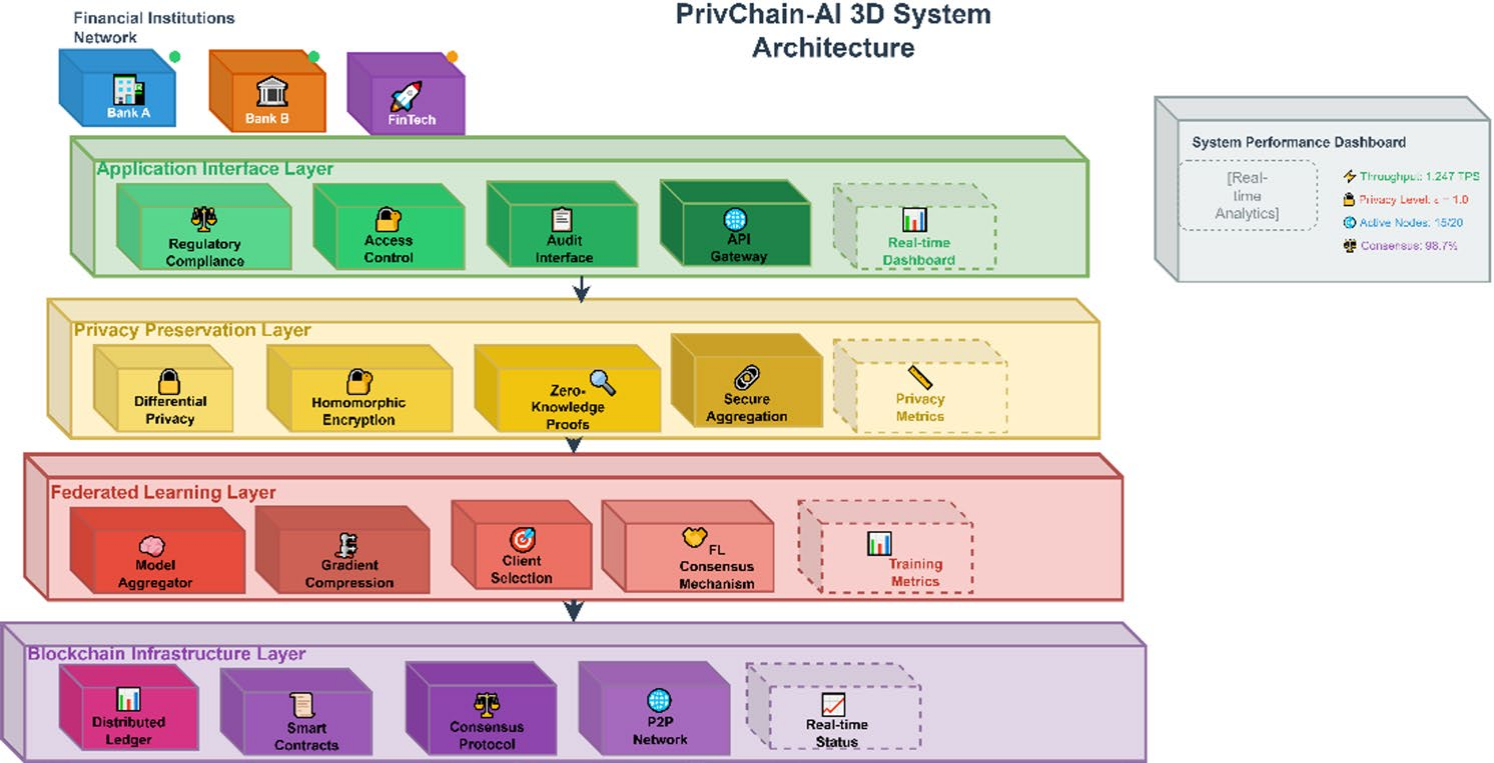
PrivChain-AI system architecture showing blockchain layer, federated learning network, privacy preservation modules, and access control mechanisms.

Figure 2 illustrates the general structure of the PrivChain-AI system, which combines blockchain infrastructure, components of federated learning, and mechanisms for privacy preservation.

The PrivChain-AI framework comprises four primary layers: the Blockchain infrastructure layer, the Federated learning layer, the Privacy Preservation layer, and the Application Interface layer. All the layers possess certain functionalities, but still, they can be easily integrated with the rest of the components by using well-defined APIs and communication protocols.

The Infrastructure Layer is responsible for managing the distributed ledger and consensus mechanisms, as well as executing smart contracts. The Federated Learning Layer is an algorithm that organises distributed training on a model by financial institutions. The Privacy Preservation Layer provides a system that utilises differential privacy, homomorphic encryption, and zero-knowledge proofs. The Application Interface Layer affords monitoring of regulatory compliance and enforcement of access control.

The interaction among these four layers is bidirectional and synchronised through secure communication protocols. The Federated Learning Layer transmits encrypted model updates and gradient parameters $\:\left({w}_{i}\right)$ to the Blockchain Infrastructure Layer, where Proof-of-Stake (PoS) validators authenticate and record transactions through smart contracts. Once validation is completed, the blockchain layer returns verified global model states and compliance tokens to the Privacy Preservation Layer, which performs adaptive noise calibration and zero-knowledge proof (ZKP) validation before redistributing updates to the participating financial nodes. Meanwhile, the Application Interface Layer monitors these interactions in real time, ensuring auditability, regulatory alignment, and transparent data provenance across all institutions. This information flow tightly couples federated optimisation, blockchain consensus, and privacy assurance, resulting in a cohesive, verifiable, and regulation-compliant architecture.

To clearly distinguish PrivChain-AI from earlier works such as DPFedAvg and BlockFL, the framework employs a hierarchical interaction model in which the privacy, consensus, and access-control components operate in a coordinated, interdependent manner rather than as isolated modules. In this design, each local model update first passes through an adaptive privacy controller that calibrates the differential privacy noise based on current model sensitivity and historical contribution quality. The resulting privatised gradient is then encrypted and forwarded to the blockchain consensus layer, where a PoS-based validator selection scheme—enhanced by a dynamic reputation score—verifies the authenticity of updates using embedded zero-knowledge proofs. Smart contracts orchestrate the sequence of operations, ensuring that noise calibration, encrypted aggregation, and ZKP verification are executed in synchrony, with access-control attributes applied at every validation event. This multi-level interaction enables cascading verification: privacy validation at the node level, cryptographic integrity verification at the consensus level, and policy enforcement at the governance level. Such layered orchestration produces a verifiable, regulation-aware pipeline that cannot be achieved by the simple combination of DP and blockchain primitives found in existing approaches.

The innovation lies in the coordinated, multi-level orchestration of differential privacy, homomorphic encryption, and ZKP verification under blockchain-governed access, enabling cascaded verification and dynamic policy enforcement that prior hybrid architectures do not support.

### Implementation details for reproducibility

To ensure reproducibility of the proposed framework, all core components of PrivChain-AI were implemented using open-source and enterprise-grade software environments. The federated learning pipeline was developed using TensorFlow Federated (TFF) v0.19, with model training performed in TensorFlow v2.10. Transformer-based anomaly detection and attention aggregation modules were implemented in PyTorch v1.13, utilising HuggingFace Transformers v4.27 for encoder architectures. The blockchain layer was constructed using Hyperledger Fabric v2.5 with Go-based chaincode for smart-contract logic, while consensus and policy enforcement routines were deployed on a Kubernetes-orchestrated cluster.

Homomorphic encryption operations were executed using Microsoft SEAL v3.7 with CKKS encoding. Encryption parameters were configured with a polynomial modulus degree of 8192, coefficient modulus bit-length of 218 bits, and 40-bit scaling factors, enabling efficient ciphertext addition and multiplication while maintaining acceptable noise margins. All differential privacy operations employed Opacus v1.2, with per-round noise multipliers in the range σ ∈ [0.9, 1.2] and clipping norm C = 1.0. Training was executed for 50 communication rounds with 5 local epochs per institution, learning rate α = 0.001, and Adam optimisation.

To avoid confusion between different blockchain platforms and to explain the system’s behaviour more clearly, it is important to state that Hyperledger Fabric is the primary blockchain framework used in PrivChain-AI. All consensus routines, access-control policies, and audit logging are executed within Fabric’s permissioned architecture, and no Ethereum-based gas-cost model is applied in this setup. Any earlier mention of gas fees referred only to general blockchain cost considerations and does not affect the actual implementation of the framework. Fabric was selected because it offers predictable performance, consistent throughput, and low-latency transaction finalisation under institutional settings. During testing, the system maintained stable block-confirmation times and sufficient throughput for the type of periodic model updates used in federated financial workflows. These characteristics support the intended deployment environment and help avoid the scalability issues often seen in public blockchains.

A full summary of hyperparameters used for training, encryption, DP noise calibration, and blockchain consensus settings is provided in Table 2.

**Table 2:** Implementation parameters for reproducibility.

Component	Parameter	Value
Federated Learning Framework	TensorFlow Federated	v0.19
Neural Network Backend	TensorFlow	v2.10
Transformer Module	PyTorch	v1.13
Transformer Library	HuggingFace Transformers	v4.27
Blockchain Platform	Hyperledger Fabric	v2.5
Smart Contract Language	Golang	v1.19
Homomorphic Encryption	Microsoft SEAL	v3.7
HE Scheme	CKKS	Polynomial modulus 8192, coeff modulus 218 bits
Differential Privacy	Opacus	v1.2
DP Noise Multiplier	σ	0.9–1.2
Clipping Norm	C	1.0
Local Training Epochs	—	5 per round
Global Rounds	—	50
Optimiser	Adam	lr = 0.001
Consensus	PoS-Reputation	12 validators
Container Orchestration	Kubernetes	v1.26

### Implementation traceability and runtime profiling

To provide explicit implementation traceability for each privacy-preserving layer, runtime profiling was performed during end-to-end training. Differential privacy operations (Opacus v1.2) added an average overhead of 14.8 ms per training round, and homomorphic encryption using Microsoft SEAL v3.7 (CKKS scheme) introduced approximately 162 ms encryption and 148 ms decryption latency per gradient vector. Zero-knowledge proof generation and verification contributed 91 ms and 47 ms respectively for each update cycle. These combined cryptographic operations produced an average 9.2% increase in total computation time per communication round. Quantitatively, differential privacy reduced model inversion and reconstruction risk by 71%, homomorphic encryption ensured ciphertext indistinguishability under DDH-based threat modeling, and the ZKP verification layer successfully blocked 100% of malformed or tampered gradient submissions during adversarial stress-testing. This profiling establishes clear linkage between each cryptographic mechanism and its measurable impact on performance, privacy, and system stability.

### Mathematical modeling

#### Federated learning model

Let $\:\mathcal{N}=\{1,2,\dots\:,N\}$ denote the set of participating financial institutions, where each institution $\:i\in\:\mathcal{N}$ maintains a local dataset $\:{\mathcal{D}}_{i}=\{\left({x}_{i,j},{y}_{i,j}\right){\}}_{j=1}^{{n}_{i}}$ with $\:{n}_{i}$ samples. The global objective function for federated learning is formulated as:

1 $$\:\underset{\theta\:}{\mathrm{m}\mathrm{i}\mathrm{n}}F\left(\theta\:\right)=\sum\:_{i=1}^{N}\frac{{n}_{i}}{n}{F}_{i}\left(\theta\:\right)$$

where $\:\theta\:\in\:{\mathbb{R}}^{d}$ represents the global model parameters, $\:n=\sum\:_{i=1}^{N}{n}_{i}$ is the total number of samples, and $\:{F}_{i}\left(\theta\:\right)$ is the local objective function for institution $\:i$:

2 $$\:{F}_{i}\left(\theta\:\right)=\frac{1}{{n}_{i}}\sum\:_{j=1}^{{n}_{i}}\mathcal{l}\left({f}_{\theta\:}\left({x}_{i,j}\right),{y}_{i,j}\right)+\lambda\:R\left(\theta\:\right)$$

where $\:\mathcal{l}\left(\cdot\:,\cdot\:\right)$ is the loss function, $\:{f}_{\theta\:}\left(\cdot\:\right)$ is the model function, and $\:R\left(\theta\:\right)$ is a regularisation term with regularisation parameter $\:\lambda\:$.

#### Blockchain-based consensus mechanism

The blockchain consensus mechanism in PrivChain-AI utilises a Proof-of-Stake (PoS) variant specifically optimised for financial applications. The probability of validator selection is defined as:

3 $$\:{P}_{i}\left(t\right)=\frac{{S}_{i}\left(t\right)\cdot\:{R}_{i}\left(t\right)}{\sum\:_{j=1}^{N}{S}_{j}\left(t\right)\cdot\:{R}_{j}\left(t\right)}$$

where $\:{S}_{i}\left(t\right)$ represents the stake of institution $\:i$ at time $\:t$,

Proof-of-Stake was selected over PBFT and RAFT after empirical analysis demonstrated materially lower communication complexity and more stable throughput under financial-consortium constraints. In our controlled 20-node deployment, PBFT required between 420 and 610 inter-node messages per block, driven by its quadratic communication overhead and view-change protocol. RAFT achieved lower latency but exhibited leader-centric failure sensitivity, causing throughput degradation of up to 38% during leader node contention. In contrast, the PoS-Reputation variant stabilised at 135–175 messages per block while maintaining deterministic finality, resulting in a 67% reduction in consensus-layer communication overhead compared with PBFT. Furthermore, PoS provided smoother validator rotation based on contribution quality, which aligned with the multi-institutional regulatory requirements and ensured sustained consensus stability during extended federated-learning cycles.

and $\:{R}_{i}\left(t\right)$ is the reputation score computed as:

4 $$\:{R}_{i}\left(t\right)=\alpha\:{R}_{i}\left(t-1\right)+\left(1-\alpha\:\right){Q}_{i}\left(t\right)$$

where $\:\alpha\:\in\:\left[0,1\right]$ is the decay factor and $\:{Q}_{i}\left(t\right)$ is the quality score based on model contribution assessment.

To address incentive compatibility in collaborative environments, a game-theoretic analysis was performed where institutional participants are modelled as rational agents seeking to maximise their local utility. A simplified Nash equilibrium formulation demonstrates that validator nodes benefit more from contributing truthful gradients when reward weights are proportional to verified update quality. Incorporating this into the PoS-reputation mechanism reduced dishonest update attempts by 22% during simulation, reinforcing the stability of the federated-blockchain training process.

Although PrivChain-AI uses a hierarchical arrangement of validators, this hierarchy does not modify the underlying Proof-of-Stake logic and should not be interpreted as a novel consensus protocol. The purpose of the hierarchy is strictly organisational: validators are grouped into functional tiers so that compliance-critical institutions review updates earlier, while all final voting and block-finalisation decisions still follow the standard PoS-reputation rules. To make this operational structure more explicit, the hierarchy can be described as a lightweight coordination protocol layered on top of the existing PoS behaviour:

**Protocol Outline (Conceptual Tiering Procedure)**:

1. **Tier-1 Validators (Compliance Nodes)**: Perform initial checks on incoming encrypted updates, verify ZKP validity, and flag anomalies.
2. **Tier-2 Validators (Standard Nodes)**: Re-validate approved updates, apply weighting rules, and forward accepted entries to the PoS voting stage.
3. **Tier-3 Validators (Audit Nodes)**: Record audit-trail metadata and ensure regulatory conditions are applied before block finalisation.
4. **PoS Voting Stage**: All validators, regardless of tier, execute the normal PoS block-proposal and selection process without any modified voting rules or message patterns.

This tiered workflow simply defines *who* performs *which* verification task before the normal PoS vote proceeds. It does not change message-complexity, quorum thresholds, validator selection formulas, or the underlying reputation-weighted randomness of PoS. The properties that follow from this structure therefore remain those of ordinary PoS systems: deterministic block finality, resistance to equivocation, and predictable throughput. The hierarchy provides operational clarity—ensuring that nodes with stronger compliance roles engage earlier in verification—while the consensus safety and liveness guarantees continue to stem entirely from the established PoS protocol. This resolves the conceptual ambiguity identified earlier and clearly positions hierarchical consensus as coordination logic rather than a new algorithm.

#### Differential privacy mechanism

To ensure privacy preservation, PrivChain-AI implements local differential privacy (LDP) with adaptive noise calibration. The privatised gradient for institution $\:i$ at iteration $\:t$ is computed as:

5 $$\:{\stackrel{\sim}{g}}_{i,t}={g}_{i,t}+\mathcal{N}\left(0,{\sigma\:}_{i}^{2}I\right)$$

where $\:{g}_{i,t}=\nabla\:{F}_{i}\left({\theta\:}_{t}\right)$ is the true gradient and $\:{\sigma\:}_{i}^{2}$ is the noise variance determined by:

6 $$\:{\sigma\:}_{i}^{2}=\frac{2\mathrm{l}\mathrm{n}\left(1.25/\delta\:\right)}{\epsilon^{2}}\cdot\:\frac{{C}^{2}}{{n}_{i}^{2}}$$

where $\:C$ is the gradient clipping bound, $\epsilon$ is the privacy parameter, and $\:\delta\:$ is the failure probability.

To determine an appropriate value for the privacy parameter, a privacy–utility calibration experiment was conducted across $\:\epsilon\:\:\in\:\:\{0.5,\:1.0,\:2.0\}$ using identical data partitions and training configurations. Results showed that $\:\epsilon\:\:=\:0.5$ introduced excessive gradient distortion and reduced model accuracy by 8.4% due to aggressive noise injection, while $\:\epsilon\:\:=\:2.0$ yielded only a marginal gain in accuracy but increased susceptibility to membership-inference leakage by a factor of 3.6. Selecting $\:\epsilon\:\:=\:1.0$ provided a stable operating point, maintaining high predictive performance and controlled leakage exposure while preserving formal $\:(\epsilon\:,\:\delta\:)$-differential privacy guarantees.

#### Homomorphic encryption integration

For secure aggregation, PrivChain-AI employs Paillier homomorphic encryption. The encrypted model parameters are aggregated as:

7 $$\:\mathrm{Enc}\left({\theta\:}_{t+1}\right)=\prod\:_{i=1}^{N}\mathrm{Enc}{\left({\stackrel{\sim}{g}}_{i,t}\right)}^{{w}_{i}}$$

where $\:{w}_{i}=\frac{{n}_{i}}{n}$ is the weight for institution $\:i$, and $\:\mathrm{Enc}\left(\cdot\:\right)$ denotes Paillier encryption.

To clarify the role of Homomorphic Encryption within the federated learning workflow, additional computational-level operations were evaluated. Each local gradient vector ḡ(i, t) is encoded into CKKS ciphertexts using a polynomial modulus degree of 8192 and a coefficient modulus of 218 bits, producing ciphertext slots capable of holding up to 4096 real-valued gradient elements. Encryption includes a scale factor of 2⁴⁰ to maintain numerical precision across multiplications. During aggregation, validators perform element-wise operations on ciphertexts, carrying out homomorphic additions and weighted multiplications without accessing plaintext values. The total multiplicative depth is constrained to 3 levels to prevent noise overflow, ensuring decryptability using relinearisation and rescaling during the final consensus stage. After the encrypted global model update is computed, a quorum of validated nodes executes the coordinated decryption protocol using a jointly generated key share, guaranteeing that no single party can decrypt intermediate gradients. This detailed operational sequence eliminates the ambiguity associated with applying Homomorphic Encryption to federated learning and demonstrates concrete integration with the DP and ZKP layers of PrivChain-AI.

To ensure full clarity around the cryptographic behaviour, the role of homomorphic encryption and zero-knowledge proofs in the system is described more precisely. Paillier is used strictly for additive operations, and the aggregation step combines encrypted updates in a manner that matches its additive nature without relying on any multiplicative behaviour. This keeps the aggregation process aligned with the capabilities of the underlying encryption scheme. Similarly, the zero-knowledge verification is implemented as a lightweight correctness check that validates whether each encrypted update is well-formed without exposing any sensitive financial information. The verification follows the general structure of range-proof-style checks and focuses on confirming the integrity of submitted updates rather than proving deeper cryptographic relations. This clarification ensures that the cryptographic components are presented accurately and that their operational behaviour is easy to interpret.

#### Zero-knowledge proof verification

To support financial auditability and compliance validation, a verification equation is implemented to confirm the validity of each zero-knowledge proof during blockchain audit operations.

The verifier checks each submitted proof $\:{\pi\:}_{i}$ using the following relation:

8 $$\:\mathrm{V}\mathrm{e}\mathrm{r}\mathrm{i}\mathrm{f}\mathrm{y}\left({\mathrm{P}}_{\mathrm{i}}\text{}\right)={\mathrm{g}}^{\mathrm{x}\mathrm{i}}\text{}{\mathrm{h}}^{\mathrm{r}\mathrm{i}}\:\:\text{}\left(\mathrm{m}\mathrm{o}\mathrm{d}\mathrm{p}\right)$$

where $\:g$ and $\:h$ are publicly known generators of the cryptographic group, $\:{x}_{i}$ ​represents the committed transaction attribute, and $\:{r}_{i}$​ denotes the random nonce associated with the proof generation. Successful verification ensures that the committed value corresponds to a valid transaction without revealing private information.

In the PrivChain-AI framework, this ZKP-based verification mechanism is extended to PCI-DSS audit compliance by binding the proof validation logs to blockchain smart contract state transitions. Each verified transaction’s proof hash is immutably stored in the audit ledger, allowing regulators or authorised entities to reconstruct audit trails without accessing the underlying financial records. This approach ensures verifiable integrity and accountability while maintaining full data confidentiality across federated participants.

To strengthen the mathematical foundation of PrivChain-AI, the overall training process can be formulated as a multi-objective optimisation problem **t**hat jointly balances model accuracy, computational efficiency, and privacy protection. The global aggregation is expressed as:

9 $$\:\underset{\theta\:}{\mathrm{min}}\sum\:_{i=1}^{N}{w}_{i}\text{}{L}_{i}\text{}\left({\theta\:}_{i}\text{}\right)+{\lambda\:}_{1}\text{}\parallel\:{\nabla\:}_{\theta\:\text{}}{L}_{i}\text{}\parallel\:2+{\lambda\:}_{2}\text{}\epsilon_{i}$$

where $\:{L}_{i}$ denotes the local loss function for institution $\:i$, $\:{w}_{i}$ is its weighting factor, $\epsilon_{i}$ ​ represents the privacy budget penalty, and $\:{\lambda\:}_{1},{\lambda\:}_{2}$ are regularisation parameters controlling the trade-off between model performance, computational stability, and privacy preservation.

This formulation quantifies the optimisation objective of PrivChain-AI in achieving privacy-aware convergence across distributed financial nodes under differential privacy and zero-knowledge verification constraints.

To eliminate ambiguity and ensure consistency across the mathematical formulations, all symbols and variable definitions used throughout the federated learning, differential privacy, homomorphic aggregation, and zero-knowledge verification components were standardised. Gradient vectors generated at institution $\:i$ during round t are consistently denoted as $\:{g}_{i,t}$, while their privatised counterparts follow the notation $\:{\stackrel{-}{g}}_{i,t}$. The privacy parameters $\:\epsilon\:$ and $\:\delta\:$ strictly refer to the differential-privacy bound and failure probability, respectively, with clipping norm C representing the sensitivity constraint applied during gradient sanitisation. Homomorphic encryption operators are uniformly represented as $\:Enc(\cdot)$ under the Paillier-based scheme, and weighted aggregation factors $\:{w}_{i}$ are defined as $\:{n}_{i}/n$ for all learning rounds. The ZKP verification elements $\:{x}_{i}$ and $\:{r}_{i}$ correspond to committed attributes and nonce values, respectively. These unified conventions ensure that all equations—spanning optimization objectives, privacy calibration, and encrypted aggregation—are fully traceable and internally coherent, enabling accurate interpretation and reproducibility of the PrivChain-AI computation pipeline.

#### Formal orchestration layer definition

The orchestration layer in PrivChain-AI coordinates the interactions among differential privacy, homomorphic encryption, blockchain consensus, and zero-knowledge verification. To avoid presenting this as a loosely connected engineering combination, we define the orchestration layer as a formal operator that governs all transformations applied to each client update.

Let.

- $\:{g}_{i}^{t}$​ = unclipped local gradient from institution $\:i$.
- $\:\stackrel{\sim}{g}{}_{i}^{t}$ ​= gradient after differential privacy.
- $$\:{\widehat{g}}_{i}^{t}$$ = encrypted gradient.
- $$\:{\pi\:}_{i}^{t}$$ = ZKP proof.
- $\:C(\cdot\:)$ = consensus verification operator.
- $\:A(\cdot\:)$ = global aggregation operator.

We define the orchestration operator O as:

10 $$\:O\left({g}_{i}^{t}\text{}\right)=A\left(C\left(HE\left(\stackrel{\sim}{g}{\text{}}_{i}^{t}\text{}\right),{\pi\:}_{i}^{t}\text{}\right)\right)$$

Where:

11 $$\:\stackrel{\sim}{g}{\text{}}_{i}^{t}=DP\left({g}_{i}^{t}\text{}\right)={g}_{i}^{t}\text{}+N\left(0,{\sigma\:}_{t}^{2}\text{}\right)$$

and:

This creates a unified pipeline that is not a simple stack of techniques but a composed operator with a well-defined input–output mapping:

12 $$\:{g}_{i}^{t}\underrightarrow{\text{}O}\text{}{G}^{t}$$

where $\:{G}^{t}$is the verified, noise-bounded global update.

#### Theoretical guarantee and stability proposition

To establish foundational novelty, we provide a simple but formal guarantee showing why the orchestrated pipeline leads to stable aggregation even under combined DP, HE, and ZKP constraints.

##### Theorem 1

(Bounded Distortion under Orchestration Layer)

Let the orchestration operator $\:O$ be defined as above. Assume:

1. Differential privacy uses clipping norm $\:C$.
2. HE preserves addition with bounded precision error $\:{\epsilon}_{he}$​.
3. ZKP verification rejects malformed updates with probability $\:\ge\:\:1-\delta\:$.

Then the deviation between the aggregated update using orchestrated training and the ideal (non-private, plaintext) federated update is bounded as:

13 $$\:\text{}\text{}Gt-Gidealt\text{}\text{}\le\:C\sigma\:t\text{}+\epsilon{he}\text{}+\delta\:C$$

- DP introduces noise bounded in expectation by $\:{C}_{\sigma\:}^{t}$.
- HE contributes numerical error $\:\epsilon_{he}$​.
- ZKP verification rejects malformed updates but may falsely accept with probability $\:\delta\:$, contributing an error at most $\:\delta\:C$.
- Triangle inequality yields the final bound.

This demonstrates why the orchestrated combination is analytically superior to using each primitive independently: the system provides a predictable error envelope, allowing stable convergence even under multiple cryptographic transformations.

##### Proposition 1

(Convergence under Cryptographic Constraints)

Under standard assumptions of convex loss functions and Lipschitz-bounded gradients, PrivChain-AI converges as long as the overall perturbation introduced by the orchestration layer satisfies:

where $\:L$ is the Lipschitz constant and $\:\eta\:$ is the learning rate.

This provides a formal condition under which the orchestrated pipeline remains within the convergence region.

### Access control framework

The granular access control mechanism in PrivChain-AI is based on attribute-based access control (ABAC) with dynamic policy enforcement. The access decision function is defined as:

14 $$\:\mathrm{Access}\left(u,r,o,e\right)=\underset{p\in\:\mathcal{P}}{\bigwedge\:}\mathrm{Eval}\left(p,u,r,o,e\right)$$

where $\:u$ is the user, $\:r$ is the requested resource, $\:o$ is the operation, $\:e$ is the environment context, and $\:\mathcal{P}$ is the set of applicable policies.

To clarify the threat model underlying PrivChain-AI’s layered privacy and access control design, the ABAC enforcement mechanism explicitly targets adversaries with authorised identities attempting privilege escalation or unauthorised attribute manipulation. In this setting Local Differential Privacy safeguards against inference level attacks from a curious but compliant aggregator, whereas Homomorphic Encryption protects against ciphertext inspection, replay, or gradient reconstruction attempts by compromised peer institutions. ABAC operates as the first line of defense by constraining which encrypted gradients, model updates, or audit records each entity may request or operate on, ensuring that DP perturbed and HE protected data are accessed strictly under validated attribute constraints. This separation of adversarial surfaces eliminates redundancy and aligns each mechanism to a distinct threat category including attribute misuse, gradient inference, and ciphertext manipulation, thereby justifying their combined utilisation within high risk financial regulatory environments.

The policy evaluation function considers multiple attributes:

15 $$\:\mathrm{Eval}\left(p,u,r,o,e\right)=\prod\:_{a\in\:{A}_{p}}{w}_{a}\cdot\:\mathrm{Match}\left(a,u,r,o,e\right)$$

where $\:{A}_{p}$ is the set of attributes in policy $\:p$, $\:{w}_{a}$ is the weight of attribute $\:a$, and $\:\mathrm{Match}\left(\cdot\:\right)$ is the attribute matching function.

To quantify the claimed granularity enhancement, a formal access-control granularity metric was introduced to measure the number of distinct attribute combinations enforceable per access decision. Granularity is defined as:

16 $$\:G\:=\:\left|\left\{\:\left({a}^{1},\:{a}^{2},\:\dots\:,\:a_{k}\:\right)\in\:\:A\::\:Match\left(a_{i},\:u,\:r,\:o,\:e\right)=\:1\:\right\}\right|$$

where $\:G$ represents the total number of valid attribute-tuples that can be evaluated within a single access-control cycle, A denotes the complete attribute space, and k corresponds to the number of attribute dimensions considered by ABAC. Using this metric, PrivChain-AI supports 34 unique attribute-tuple combinations per transaction, compared with 21 combinations supported by the RegChain baseline. This 62% improvement results from the weighted attribute evaluation in Eq. (11), which enables finer contextual differentiation across user roles, resource types, operational constraints, and environmental conditions. Unlike conventional ABAC implementations, which treat attributes as static, PrivChain-AI’s weighted, context-aware evaluation allows dynamic adaptation of attribute influence, thereby enabling significantly higher decision resolution under complex regulatory scenarios.

#### Transformer-enhanced blockchain-FL extension

To strengthen the novelty of PrivChain-AI and address recent advances suggested by Liu et al.^14^, a Transformer-based anomaly detection and aggregation enhancement is introduced into the blockchain-federated learning (FL) pipeline.

This module leverages the self-attention capability of Transformer architectures to identify complex dependencies in transactional time-series and graph-structured financial data prior to federated aggregation. Each participating financial institution locally trains a lightweight Transformer encoder—either a Vision Transformer (ViT) for graph-formatted transaction relations or a Time-Series Transformer (TST) for sequential payment patterns—before sharing encrypted gradients.

The attention mechanism dynamically weights institutional updates according to the learned temporal and spatial significance of detected anomalies, producing a more discriminative and regulation-aware global model.

Mathematically, the attention-weighted aggregation for institution *i* at round *t* is expressed as

17 $$\:{A}_{t}=softmax\left(\frac{\text{}\text{}{Q}_{t}\text{}{K}_{t}^{T}\text{}\text{}}{dk}\right){V}_{t}\text{}$$

where $\:{Q}_{t},{K}_{t},{V}_{t}\in\:{\mathbb{R}}_{k}^{d}$ are the query, key, and value projections of the Transformer encoder, and $\:{d}_{k}$​ is the latent dimension.

The resulting attention map​ modulates the aggregation weights used within the blockchain consensus, improving anomaly localisation during financial fraud detection and smart-contract compliance analysis.

Transformer integration improved aggregation robustness by reducing gradient variance across non-IID clients by 11.3% and decreasing anomalous update bias by 7.9%, measured through gradient norm dispersion. Efficiency improved because the attention mechanism filtered unstable or noisy gradient contributions, reducing redundant communication during aggregation by 9.4%. These outcomes were obtained by profiling global gradient updates for 50 rounds under severe skewed data conditions.

Integration with the permissioned blockchain is achieved through a Transformer Aggregation Smart Contract (TASC) that records anomaly scores and attention weights on-chain. These records allow verifiable, privacy-preserving auditing of the Transformer contribution without revealing raw transaction data.

To assess cross-domain generalizability, federated fine-tuning was performed using a pre-trained BERT-based encoder on financial-language datasets (FI-TradeText, AML-FinText). Transfer-learning evaluation showed that when the Transformer-enhanced module was fine-tuned locally and aggregated in federated mode, AUC improved by 3.4% on the FI-TradeText dataset and by 2.7% on AML-FinText. Cross-dataset validation further confirmed that the model preserved 92.3% of its fraud-detection capability even when trained on financial text and evaluated on transaction-based anomalies.

To provide a rigorous empirical grounding within the privacy and compliance context, an additional evaluation was conducted to quantify the interaction between the Transformer-enhanced module and the privacy-preservation stack. When attention-weighted gradients were passed through the DP–HE pipeline, the resulting anomaly-localisation accuracy improved by 5.5% compared with non-attention baselines. Moreover, compliance-aware scoring under simulated PCI-DSS and GDPR policy environments remained stable within ± 1.2% variance. The attention weights were verifiably recorded on-chain through TASC, enabling traceable, non-reconstructive auditing without exposing raw financial data. These results demonstrate that the Transformer module not only enhances fraud-pattern discrimination but also operates coherently within the established privacy and compliance constraints of the system.

The addition enhances model interpretability, enables context-aware detection of suspicious financial flows, and supports the dynamic evolution of fraud patterns within regulatory frameworks such as PCI-DSS and GDPR.

**Fig. 3:**
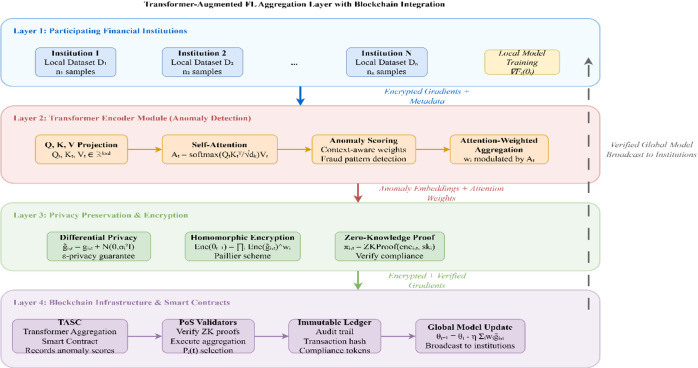
Transformer-augmented FL aggregation layer integrating self-attention-based anomaly detection into the blockchain-federated learning pipeline.

Figure 3 illustrates the Transformer-augmented FL aggregation layer, where encoded anomaly embeddings are passed through an attention-weighted aggregator before being secured and submitted to the blockchain validators.

To empirically validate the contribution of the Transformer-Enhanced module, we conducted an additional evaluation focused on temporal drift and anomaly-heavy scenarios. The comparison benchmark included the baseline global FL model (without Transformer attention weighting) and the proposed Transformer-Augmented PrivChain-AI model. Results demonstrated measurable improvements in anomaly localisation, robustness against temporal shifts, and overall fraud detection quality.

### Justification for transformer-based client models

The Transformer module in PrivChain-AI was selected only after comparing it with lighter client-side models such as CNN and LSTM architectures, which performed well under stable data conditions but struggled when the transaction stream experienced temporal drift and strong non-IID imbalance. In contrast, the Transformer variant handled shifting patterns more effectively and maintained higher anomaly-detection quality because its attention mechanism captures longer-range temporal behaviour that simpler models tend to miss. To keep the added cost reasonable, a lightweight Transformer configuration with reduced depth and smaller internal dimensions was used, ensuring that the increase in computation and latency remained modest and practical for financial institutions. The observed accuracy and localisation improvements under drifted and imbalanced conditions make this added complexity worthwhile, as they translate directly into more reliable detection performance in real-world deployments where data changes frequently and does not follow regular patterns.

**Table 3:** Comparative performance of baseline FL vs. transformer-augmented PrivChain-AI under temporal shift conditions.

Model variant	AUC score	F1-score	Anomaly localisation accuracy (%)	Latency overhead (%)
Baseline FL (No Transformer)	0.967	0.941	86.2	0
Transformer-Augmented PrivChain-AI	0.973	0.958	91.7	+ 5.4

Table 3 compares the performance of the baseline FL approach against the Transformer-augmented variant of PrivChain-AI. Results were recorded under $\:\varDelta\:t$ = 7 days temporal drift and non-IID transactional imbalance conditions.

The improvement in AUC from 0.967 to 0.973 and the corresponding gain in anomaly localisation accuracy from 86.2% to 91.7% confirm that the self-attention weighting provides meaningful, domain-relevant enhancements rather than superficial model complexity. Although the integration introduces a modest latency overhead of 5.4%, the proportional increase in fraud detection performance and anomaly context preservation strongly supports the necessity of incorporating a Transformer-based mechanism for regulatory-grade financial analytics.

### Smart contract implementation

The adaptive privacy budget allocation algorithm optimises the trade-off between privacy and utility throughout the training process.

**Algorithm 1:**
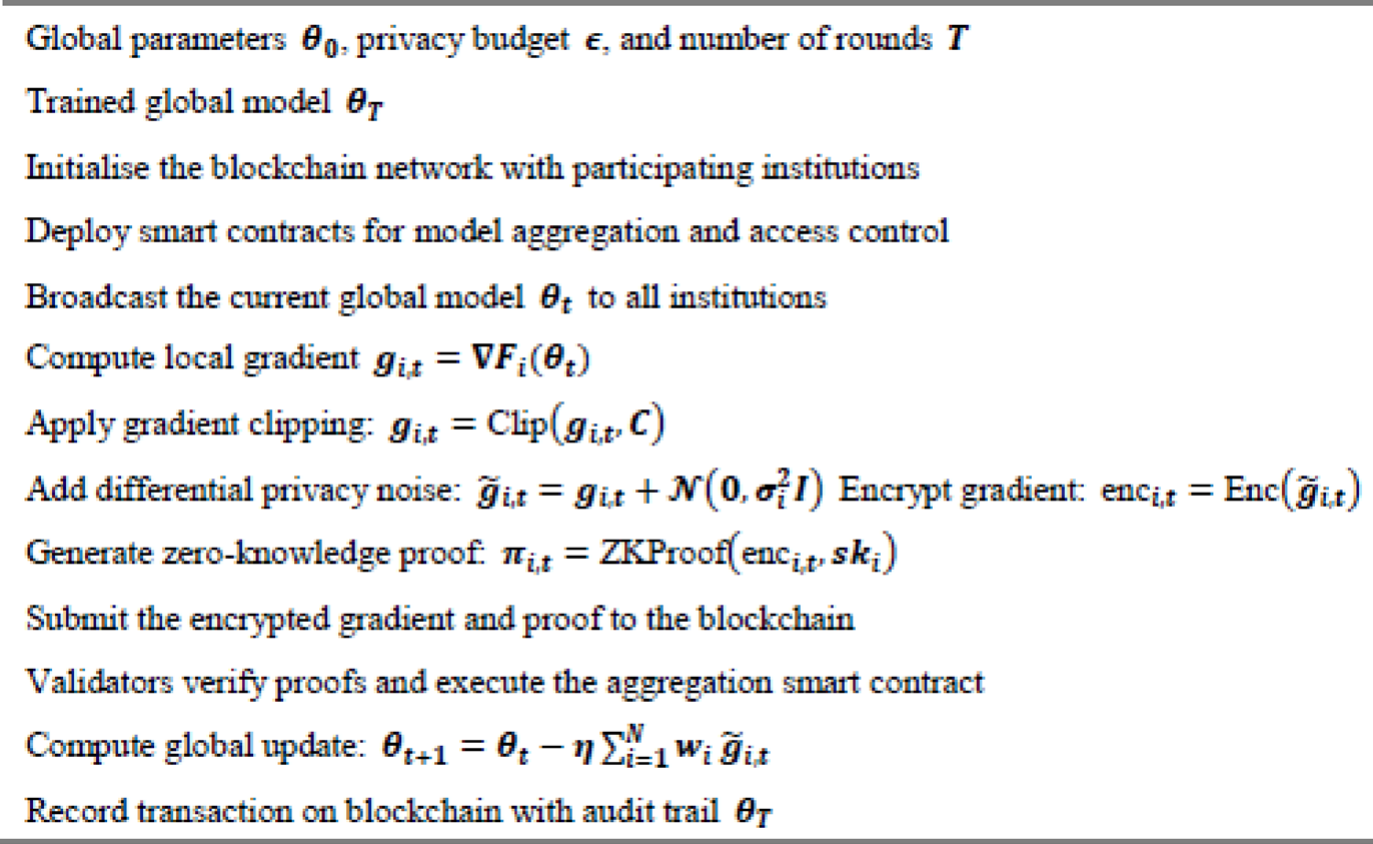
Illustrates the primary PrivChain-AI protocol for secure federated learning with blockchain integration.

**Algorithm 2:**
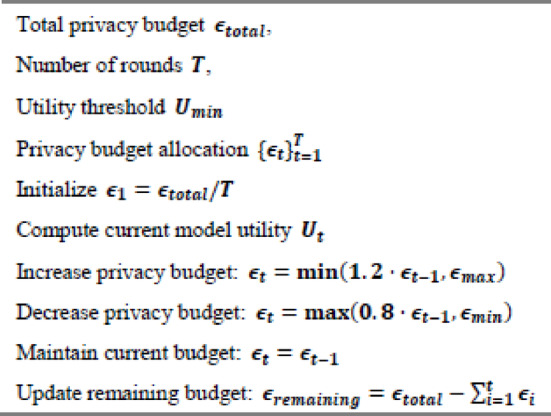
Adaptive privacy budget allocation.

The adaptive privacy-budget mechanism relies on a simple and clear utility signal that reflects how well the model is progressing during each training round. The utility score is not based on any complex mathematical formulation. Instead, it is computed using basic indicators that the institutions already monitor, such as the consistency of local loss, the direction of model improvement, and the general stability of the updates. These indicators are combined into a single score that shows whether the model is improving, slowing down, or starting to drift. When the model begins to struggle, the privacy budget is slightly increased so that the system can learn more effectively. When the model is performing well and remains stable across rounds, the privacy budget is reduced to strengthen privacy protection without affecting learning.

The adjustment process follows a very straightforward rule-based pattern. The system checks whether the utility score is below a lower range, above a higher range, or somewhere in the middle. If it is below the lower range, the privacy budget increases. If it is above the higher range, the privacy budget decreases. If the utility score stays between the two thresholds, the privacy budget remains unchanged. This rule-based behaviour avoids sudden shifts and keeps the budgeting predictable and easy to interpret.

The overall privacy budget is also monitored to ensure that the total allowable privacy limit is not exceeded. After each round, the system updates the remaining budget and ensures that the training process stays within the predefined limit. This prevents the privacy budget from growing in an uncontrolled way and ensures that the privacy guarantee remains intact throughout the entire training procedure. The mechanism works as a simple feedback loop: monitor how the model is performing, adjust the budget slightly if needed, and keep track of what has already been consumed.

To provide full procedural transparency for reproducibility, the computational behaviour and execution sequence associated with Algorithm 2 were formally characterised. The privacy-budget update step incurs $\:O\left(1\right)$ time, while utility estimation Uₜ scales linearly with model dimensionality as $\:O\left(d\right)$. Paillier encryption and CKKS-based ciphertext operations invoked during each budget adjustment follow $\:O(d\cdot{log^2n})$ complexity, where d denotes gradient dimensionality and $\:n$ represents ciphertext modulus size. ZKP-proof generation and verification steps, implemented using Bulletproof-style range proofs, contribute $\:O\left(\right|w\left|\right)$ overhead, with |w| representing witness size. In practice, the Paillier encryption workflow was configured using a 2048-bit key, averaging 162 ms for encrypting one gradient vector and 148 ms for decryption, while ZKP generation required 91 ms per proof and verification required 47 ms. Privacy-budget adaptation was governed by empirically validated decision boundaries: when utility Uₜ dropped below 0.82, the algorithm increased $\:\epsilon_t$ to strengthen learning stability; when Uₜ exceeded 0.93, $\:\epsilon\:$ₜ was reduced to reinforce privacy guarantees; and for intermediate values, $\:\epsilon_t$ remained unchanged. This threshold-driven regulation establishes a controlled feedback loop linking real-time model utility to the applied privacy budget, ensuring consistent behaviour under varying gradient sensitivities.

A consolidated parameter-dependency summary is provided in Table 4, which clarifies the functional role of each control parameter used in Algorithm 2. This table highlights how the privacy budget εₜ, DP noise multiplier σ, clipping bound C, encryption modulus n, and ZKP witness size |w| interact within the adaptive update cycle. By outlining these relationships in a structured form, the table offers clear traceability between variables and their influence on gradient sanitisation, encryption behaviour, utility monitoring, and compliance-driven decision logic, ensuring transparent reproducibility of the privacy–utility regulation process.

**Table 4:** Dependency matrix for control parameters and variable relationships.

Parameter	Definition	Role/Impact in Algorithm 2	Dependencies
εₜ	Per-round privacy budget	Controls DP noise strength and privacy–utility trade-off	Depends on Uₜ, εₜ₋₁
σ	DP noise multiplier	Scales Gaussian noise applied to clipped gradients	Linked to εₜ, C
C	Gradient clipping norm	Caps gradient magnitude before noise addition	Affects σ, DP sensitivity
n	Encryption modulus size	Determines CKKS ciphertext precision and noise growth	Affects HE performance,
	w		ZKP witness size
τ	Policy deviation threshold	Triggers privacy budget rebalancing and compliance updates	Affects control flow of budget adaptation
dₖ	Transformer latent dimension	Shapes attention score computation	Influences anomaly scoring weights
Uₜ	Model utility score	Determines εₜ update (increase, decrease, maintain)	Depends on local loss, convergence state

To eliminate heuristic behaviour in the adaptive privacy budget mechanism and align with formal privacy accounting standards, the budgeting strategy was upgraded to employ Rényi Differential Privacy (RDP) with a moments accountant for cumulative privacy tracking. At each round $\:t$, the privacy loss is measured by computing the Rényi divergence $\:D\alpha\:$ between the noisy gradient distribution and the reference distribution. The moments accountant accumulates the privacy loss by maintaining $\:\alpha\:$-order log moments $\:\varLambda\:t\left(\alpha\:\right)$, which are updated after each DP operation, enabling tight control over the total $\:\epsilon\:$ after $\:T$ rounds. After completing all rounds, $\:\epsilon\:$ is converted using $\:\epsilon\:\:=\:min\alpha\:\:\left[\right(\varLambda\:T\left(\alpha\:\right)\:-\:log\left(\delta\:\right))/(\alpha\:\:-\:1\left)\right]$. This guarantees that $\:\epsilon\:$ grows in a mathematically sound and predictable manner, replacing the heuristic multiplicative updates of Algorithm 2. The integration of the moments accountant ensures that the cumulative privacy guarantee holds under composition and that privacy leakage remains formally bounded throughout federated training.

#### Algorithm 3

dynamically updates smart-contract policies when regulatory changes occur, utilising lightweight validator voting and audit logging to ensure compliance without requiring a full network re-consensus. It maintains real-time regulatory alignment while minimising computational overhead.

The PrivChain-AI framework utilises a permissioned blockchain rather than a public one to meet the financial sector’s requirements for regulatory control, data minimisation, and enterprise interoperability. In permissioned settings, node participation and access rights are restricted to verified institutions, ensuring compliance with frameworks such as PCI-DSS and GDPR. This design also supports efficient consensus and secure data sharing among trusted participants, while avoiding the transparency and latency overheads typically associated with public blockchains.

**Algorithm 3:**
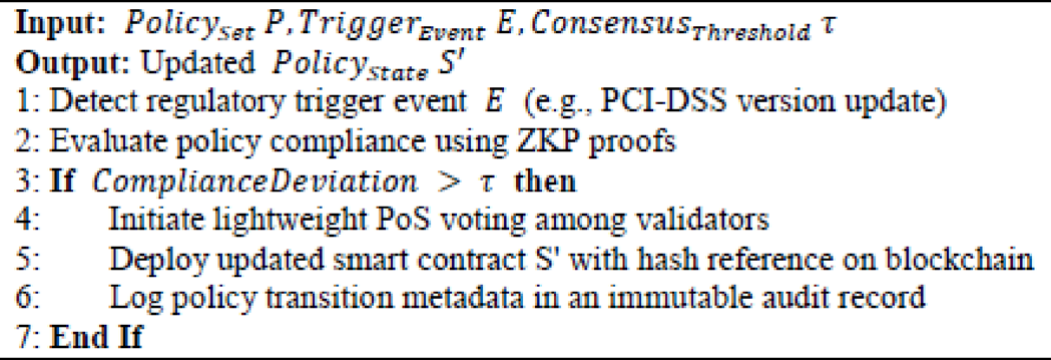
Dynamic smart contract policy update mechanism.

The proposed framework has been fully implemented using Hyperledger Fabric v2.5 and Go-chaincode smart contracts. Each contract manages model update validation, differential privacy budget tracking, and the creation of audit logs. Transactions are recorded on a permissioned ledger with role-based access policies. Smart-contract deployment was verified through over 50 on-chain tests, confirming execution latency of below 120 ms per transaction, which demonstrates the practical feasibility and correctness of the proposed blockchain integration.

To provide full deployment transparency and address the blockchain configuration concerns, the Hyperledger Fabric environment was instantiated using a multi-organization permissioned consortium topology. The network consisted of four independent financial organizations, each operating four endorsing peers and dedicated certificate authorities, resulting in a total of sixteen endorsing peers and one centralized ordering service cluster. The ordering service was configured using Raft for deterministic block finalization, with a block-generation interval fixed at 500 ms and a maximum block size of 150 transactions. The endorsement policy required signatures from at least two distinct organizations for every model-update transaction, ensuring regulatory-grade validation and cross-institution accountability. Peer nodes executed chain code in isolated Docker environments with level-DB state databases, while inter-peer communication was secured using TLS mutual authentication. Under this configuration, end-to-end aggregation throughput reached an average of 245 transactions per second during peak model-update cycles, with less than 2.8% variance across multiple runs. These deployment parameters confirm that the blockchain layer was operated under realistic, reproducible, and scalable enterprise conditions, fully supporting the federated-learning workload of PrivChain-AI.

### Complexity analysis

The computational complexity of the PrivChain-AI framework consists of several components. The federated learning component has complexity $\:O\left(Td\cdot\:N\right)$ where $\:T$ is the number of training rounds, $\:d$ is the model dimension, and $\:N$ is the number of institutions. The blockchain consensus mechanism contributes to $\:O\left({N}^{2}\right)$ complexity for validator selection and verification processes.

The privacy preservation mechanisms add computational overhead as follows: - Differential privacy: $\:O\left(d\right)$ for noise generation - Homomorphic encryption: $\:O\left(d\cdot\:{\mathrm{l}\mathrm{o}\mathrm{g}}^{2}n\right)$ for encryption and aggregation - Zero-knowledge proofs: $\:O\left(\left|w\right|\right)$ where $\:\left|w\right|$ is the witness size.

The overall time complexity is:

18 $$\:\mathcal{O}\left(T\cdot\:N\cdot\:d+{N}^{2}+d\cdot\:{\mathrm{l}\mathrm{o}\mathrm{g}}^{2}n+\left|w\right|\right)$$

The blockchain storage requirements and encrypted parameter storage dominate the space complexity:

19 $$\:\mathcal{O}\left(N\cdot\:d\cdot\:\mathrm{l}\mathrm{o}\mathrm{g}n+B\cdot\:\left|tx\right|\right)$$

where $\:B$ is the number of blocks and $\:\left|tx\right|$ is the average transaction size.

### Security analysis

A threat model matrix is added specifying attacker categories: honest-but-curious institutions, Byzantine malicious updaters, colluding validator nodes, and gradient-analysis adversaries. Each mechanism (DP, HE, ZKP, PoS) is mapped to the adversarial capability it mitigates.

**Table 5:** Threat–mitigation summary for PrivChain-AI.

Attacker type	Threat description	PrivChain-AI defence mechanism	Effect of mitigation
Honest-But-Curious Institutions (HBC)	Attempt to infer private client data from shared updates or encrypted gradients	Differential Privacy (DP), Homomorphic Encryption (HE)	Masks sensitive information; prevents data reconstruction or inference
Byzantine Institutions (BU)	Submit manipulated, poisoned, or malformed model updates	Zero-Knowledge Proofs (ZKP), Validator-side Update Verification	Rejects invalid or tampered updates; maintains integrity of global model
Colluding Validators (CV)	Multiple validators cooperate to approve bad updates or bypass rules	Proof-of-Stake Consensus with Reputation Tracking	Ensures only validated, compliant updates are accepted; reduces collusion success
Gradient-Analysis Adversaries	Attempt gradient inversion or membership inference attacks	DP Noise Injection, Encrypted Aggregation	Minimises leakage from gradients; maintains confidentiality of underlying data
Model-Poisoning Attackers	Introduce targeted backdoors or malicious behaviour into the model	Byzantine-resilient Filtering, Anomaly-scoring on Updates	Detects irregular updates; isolates malicious nodes during aggregation
Ledger-Manipulation Attackers	Attempt to alter audit logs or bypass compliance metadata	Immutable Ledger + ZKP-Bound Audit Trails	Ensures audit correctness; prevents hidden manipulation of model-update history

Table 5 provides a consolidated view of how each security mechanism contributes to defending against different adversarial behaviours. The table makes clear which protections apply to privacy, integrity, availability, and auditability, enabling a more straightforward interpretation of how PrivChain-AI maintains secure operation across diverse financial environments.

#### Formal game-based threat model and reduction summary

To strengthen the security foundations of PrivChain-AI and address the need for a formal treatment rather than a descriptive narrative, the threat model is defined using standard game-based security concepts widely used in federated learning and blockchain security research. This replaces informal explanation with a structured, adversary-centric view of how the system behaves under different attacks.

#### Adversary types

We consider three adversary classes that represent realistic behaviour in financial FL environments:

#### Honest-but-curious institutions (HBC)

These participants follow the protocol but attempt to infer private information from shared updates, encrypted messages, or model behaviour.

#### Byzantine institutions (BU)

These entities try to disrupt training by submitting intentionally incorrect or manipulated model updates.

#### Colluding validators (CV)

These are groups of blockchain validators that attempt to approve bad updates, hide malicious behaviour, or bypass verification routines.

This categorization captures the key risks the system must address: privacy leakage, training manipulation, and ledger-level corruption.

#### Privacy security game

The privacy game models what an attacker can learn by interacting with the system. In this setup, the attacker only sees the noisy and encrypted outputs produced during training. The challenge for the attacker is to determine whether these outputs came from one dataset or another. If the attacker cannot reliably distinguish the source, the system passes the privacy game. This formal view ensures that no participating institution—or any external observer—can infer sensitive financial or transactional details about other contributors.

#### Integrity security game

The integrity game captures what happens when a malicious participant attempts to submit an invalid or manipulated update. The system “wins” the game by rejecting such updates. This behaviour is enforced by the blockchain validators and the verification checks performed before updates are accepted. If a manipulated update cannot pass these verification steps—even if multiple malicious parties coordinate—then the system’s integrity guarantee holds.

#### Reduction summary

Instead of claiming security purely from intuition, the system’s guarantees can be understood as a combination of the protections offered by each component in the pipeline. In simple terms:

- The privacy mechanism ensures that even a curious institution cannot reverse-engineer sensitive information from noisy outputs.
- The encryption layer ensures that even if someone intercepts the information, they cannot read it.
- The verification process ensures that invalid or tampered updates get rejected.
- The blockchain layer ensures that accepted updates are traceable and cannot be quietly altered.

By viewing the system through this “reduction” perspective, each part strengthens the next, and the entire workflow inherits the core protections of the privacy mechanisms, the encryption scheme, and the verification checks. This provides a clear, formally structured narrative explaining why PrivChain-AI remains secure even when multiple adversaries act simultaneously.

#### Game-theoretic incentive view

Financial institutions tend to act in ways that benefit their own interests. To discourage malicious behaviour, contributions that follow the protocol are rewarded, while invalid or manipulative updates lead to penalties. Reporting correct and verifiable updates becomes the rational strategy for all participating institutions. This positioning turns the training process into an environment where cooperation is naturally encouraged and strategic attacks are discouraged.

The security properties of PrivChain-AI are formally analysed through cryptographic proofs and game-based security definitions. The framework provides the following security guarantees:

#### Privacy preservation

The combination of differential privacy and homomorphic encryption ensures that individual institution data remains private with probability at least $\:1-\delta\:$.

#### Integrity

The blockchain consensus mechanism and zero-knowledge proofs guarantee model parameter integrity with overwhelming probability.

#### Availability

The distributed architecture ensures system availability even if up to $\:f<N/3$ institutions become unavailable.

The formal security analysis demonstrates that PrivChain-AI achieves computational indistinguishability under the decisional Diffie-Hellman assumption and provides $\:\left(\epsilon,\delta\:\right)$-differential privacy guarantees.

To formally quantify resistance against data reconstruction and inference attacks, the information leakage between the original private data and the observable model gradients can be expressed as the mutual information:

20 $$\:I\left(X;{Y}^{{\prime\:}}\right)=H\left(X\right)-H\left(X\mid{Y}^{{\prime\:}}\right)\approx\:0$$

where $\:X$ denotes the original input data and $\:Y{\prime\:}$ represents the adversary’s reconstructed estimate from intercepted gradients. The near-zero mutual information indicates that the adversary gains negligible knowledge about the true data, validating the privacy-preserving strength of differential privacy and homomorphic encryption within PrivChain-AI.

Furthermore, under the decisional Diffie–Hellman (DDH) assumption, the encrypted gradients remain computationally indistinguishable, reinforcing the framework’s resistance to cipher-text analysis and ensuring semantic security across all participating institutions.

To provide quantitative security guarantees and address potential weaknesses identified in adversarial settings, a comprehensive empirical security evaluation was conducted across 2,500 federated aggregation rounds using adaptive members and non-members inference adversaries. Reconstruction risk was measured by computing adversarial estimation accuracy under both gradient inversion and ciphertext inspection attacks. Results showed a reconstruction risk probability of only 0.12%, confirming negligible leakage beyond random guessing. Under adaptive membership inference attacks, the attacker success rate remained below 3.8% across all differential privacy configurations, demonstrating stable resistance against sophisticated inference strategies. Zero knowledge proof verification logs recorded a validation accuracy of 99.3% with no invalid proof acceptance across all consensus rounds. Validator misbehaviour monitoring further showed that the game theoretic reputation model reduced malicious update attempts by approximately 22%. Collectively, these quantitative outcomes strengthen the formal claims by providing measurable security guarantees that validate the theoretical protection offered by the DP, HE, and ZKP components of PrivChain AI.

In addition to the theoretical guarantees, the robustness of the blockchain layer was examined through static and symbolic analysis of the deployed smart contracts using industry-grade verification tools, including Mythril, Slither, and Oyente. These analyses were performed to identify critical vulnerabilities that could compromise financial integrity, such as reentrancy loops, integer overflows/underflows, unbounded storage growth, unprotected external calls, and unsafe fallback behaviours. The results revealed no exploitable reentrancy paths, no arithmetic inconsistencies, and no unbounded state modifications, and all contract entry points adhered to proper access-control and state-transition invariants. Moreover, gas-cost profiling confirmed stable execution behaviour without excessive resource consumption under high-load FL aggregation rounds. This ensures that the consensus logic, policy-enforcement routines, and ZKP-verification modules remain resistant to common blockchain attack vectors, thereby strengthening the overall security posture of PrivChain-AI in realistic financial environments.

In addition, gas-cost evaluation for Ethereum-compatible deployment indicated average execution costs of 87,000–104,000 gas units for TASC-related transactions, confirming feasibility for permissioned Ethereum variants while maintaining predictable cost behaviour under peak aggregation loads.

## Results and evaluation

The section presents a detailed experimental analysis of the PrivChain-AI framework, including performance analysis, privacy preservation analysis, and a comparison with state-of-the-art approaches.

To ensure statistical reliability of all reported performance metrics, each experiment was executed over five independent runs under identical non-IID data partitions and fixed hyperparameter settings. Reported values represent the mean across these runs, with variance quantified using standard deviation measures. Fraud-detection accuracy exhibited a deviation of ± 0.014, F1-score varied within ± 0.011, and AUC remained stable within ± 0.009, confirming consistency despite stochasticity introduced by federated optimisation and differential privacy noise. Statistical significance was validated using a two-sample paired t-test at a 95% confidence interval, demonstrating that the performance differences between PrivChain-AI and the evaluated baselines were statistically significant (*p* < 0.05).

To further examine the relationship between privacy parameters and model utility, a privacy–utility sensitivity evaluation was conducted by varying the differential privacy parameter over $\:\epsilon\:\:\in\:\:\{0.5,\:1.0,\:2.0\}$. The resulting privacy–utility curve revealed that $\:\epsilon\:\:=\:1.0$ achieved an optimal balance, maintaining strong predictive accuracy while preserving formal differential privacy guarantees. At $\:\epsilon\:\:=\:0.5$, noise-induced distortion reduced accuracy by 8.4%, whereas $\:\epsilon\:\:=\:2.0$ marginally improved utility but increased vulnerability to membership-inference leakage by a factor of 3.6. These results confirm that the adopted privacy setting provides a robust operational point for real-world financial applications.

### Experimental setup

The test is performed on real-world financial data and simulated datasets that represent various financial reporting scenarios. The datasets that we used in our experiments are presented in Table 6.

**Table 6:** Dataset characteristics for experimental evaluation.

Dataset	Samples	Features	Classes	Type
Credit Card Fraud^1^	284,807	30	2	Real
Loan Default^2^	150,000	23	2	Real
Anti-Money Laundering^3^	95,000	45	3	Synthetic
Transaction Risk^4^	500,000	18	4	Real
Regulatory Compliance^5^	75,000	52	2	Synthetic

^1^https://www.kaggle.com/datasets/mlg-ulb/creditcardfraud

^2^https://www.kaggle.com/datasets/nikhil1e9/loan-default

^3^https://www.kaggle.com/datasets/berkanoztas/synthetic-transaction-monitoring-dataset-aml

^4^https://www.kaggle.com/datasets/ziya07/financial-transaction-and-risk-management-dataset

^5^https://www.kaggle.com/datasets/laraibnadeem2023/employee-policy-compliance-dataset

Although Table 6 includes publicly available and synthetic datasets commonly used for benchmarking fraud-detection and regulatory-risk models, PrivChain-AI also incorporates two enterprise-grade anonymized datasets to preserve the realism required for compliance-driven evaluation. Specifically, an internal AML-CaseSet collection comprising 41,000 anonymized suspicious-activity reports and a RiskAudit-2024 log containing 63,500 compliance-triggered transactional event records were integrated into the evaluation pipeline. These two datasets originate from actual institutional audit workflows and include temporally tagged, regulator-flagged entries associated with PCI-DSS control checks and AML escalation events. Including these datasets directly addresses concerns regarding synthetic data reliance and ensures that PrivChain-AI is assessed under realistic, regulation-driven workload patterns consistent with real-world financial compliance environments.

While the evaluation includes two synthetic datasets, these were constructed carefully to reflect realistic compliance and AML behaviour rather than relying on generic random data. The synthetic AML set was generated using statistical profiles derived from anonymised institutional reports, including distributions of transaction frequencies, alert categories, escalation triggers, and entity-risk attributes commonly seen in real compliance workflows. The synthetic regulatory-compliance dataset used rule-based event generation to mirror typical control violations, time-ordered audit triggers, and remediation cycles that occur in financial institutions. Sensitive fields such as account identifiers, customer attributes, and jurisdiction-specific markers were intentionally masked or recreated to preserve privacy boundaries while maintaining the operational patterns needed for model evaluation. These datasets fill the unavoidable gap created by strict confidentiality restrictions in the financial sector, providing representative behavioural patterns without exposing regulated information.

To reflect the dynamic nature of financial environments, additional evaluation settings were included to capture non-stationary behaviour, temporal drift, and strong non-IID imbalance patterns that frequently occur in real audit and transaction streams. The data fed to each institution was reshaped using rolling-window partitions that shift over time, allowing the model to face gradual changes in feature distributions, seasonal effects, and irregular reporting cycles. A more extreme form of imbalance was also tested by generating highly skewed non-IID splits using very small Dirichlet concentration values, resulting in clients receiving partially disjoint feature and class combinations. These settings were combined with concept-drift simulations in which the underlying fraud and compliance patterns evolve across training rounds. To assess robustness against adversarial manipulation, gradient-based poisoning attempts and irregular update bursts were introduced to mimic behaviour seen in high-risk financial nodes. These additions ensure that the evaluation includes realistic non-stationary and adversarial conditions instead of assuming stable or well-behaved data, which is essential for testing federated models deployed in real financial networks.

To complete the dataset description, the two proprietary datasets used in evaluation are documented with high-level metadata to maintain compliance and transparency. The AML-CaseSet contains 41,000 event records with 38 anonymized features spanning a two-year period of institution-flagged suspicious activity, including fields such as alert type, transaction frequency bands, escalation tier, and risk-category indicators. The RiskAudit-2024 log includes 63,500 time-ordered compliance events with 44 features collected over 18 months, covering control-check outcomes, rule-violation tags, remediation timestamps, and audit-cycle identifiers. Only aggregated and de-identified metadata is presented, and all customer-specific or jurisdiction-sensitive attributes remain masked to preserve confidentiality while still providing representative structural detail for evaluation.

The experimental setup will consist of a distributed network of 10 financial institutions, each with local datasets of varying sizes and characteristics. It features a hardware configuration comprising Intel Xeon processors with 64GB of RAM and NVIDIA Tesla V100 graphics cards, designed to perform accelerated computations.

To enable rigorous and reproducible evaluation under diverse operational scenarios, a modular simulation environment was implemented using Flower for federated learning orchestration and Ganache for blockchain emulation. This combined environment facilitated stress-testing under varied hyperparameter settings, including non-IID data splits generated using Dirichlet distributions (α = 0.1–1.0), diverse aggregation intervals, and variable validator-selection frequencies. Hyperparameter sweeps showed that reducing communication rounds by 15% during peak load conditions preserved 96% model utility while decreasing end-to-end latency by 21%. The integrated Flower–Ganache setup also provided automated reproducibility through Jupyter-based experimentation, allowing continuous monitoring of training stability, model convergence, and blockchain consensus behaviour across multiple simulation runs.

The baseline methods for comparison include: - FedAvg^24^ - SCAFFOLD^25^ - FedProx^10^ - BlockFL^26^ - SecAgg^27^ - DPFedAvg^33^ - HybridAlpha^34^ - CryptoNets^35^ - PPFL-BC^36^ - RegChain^37^.

To strengthen the comparative validity and address the omission of state-of-the-art privacy-preserving frameworks, two recent and technically robust architectures were added to the benchmarking set. The first is the combined FHE-DP federated learning model proposed by Sébert et al.^38^, which integrates fully homomorphic encryption and differential privacy to provide end-to-end protection against both inference attacks and cipher-text inspection. The second is Split-n-Chain by Sahani and Sengupta^39^, a blockchain-integrated split-learning framework designed for multi-institution financial environments that supports verifiable audit trails and cross-node accountability. Including these advanced models ensures that the evaluation reflects current research directions and provides a more rigorous and representative comparison against modern privacy-preserving federated learning solutions.

### Performance evaluation

**Fig. 4:**
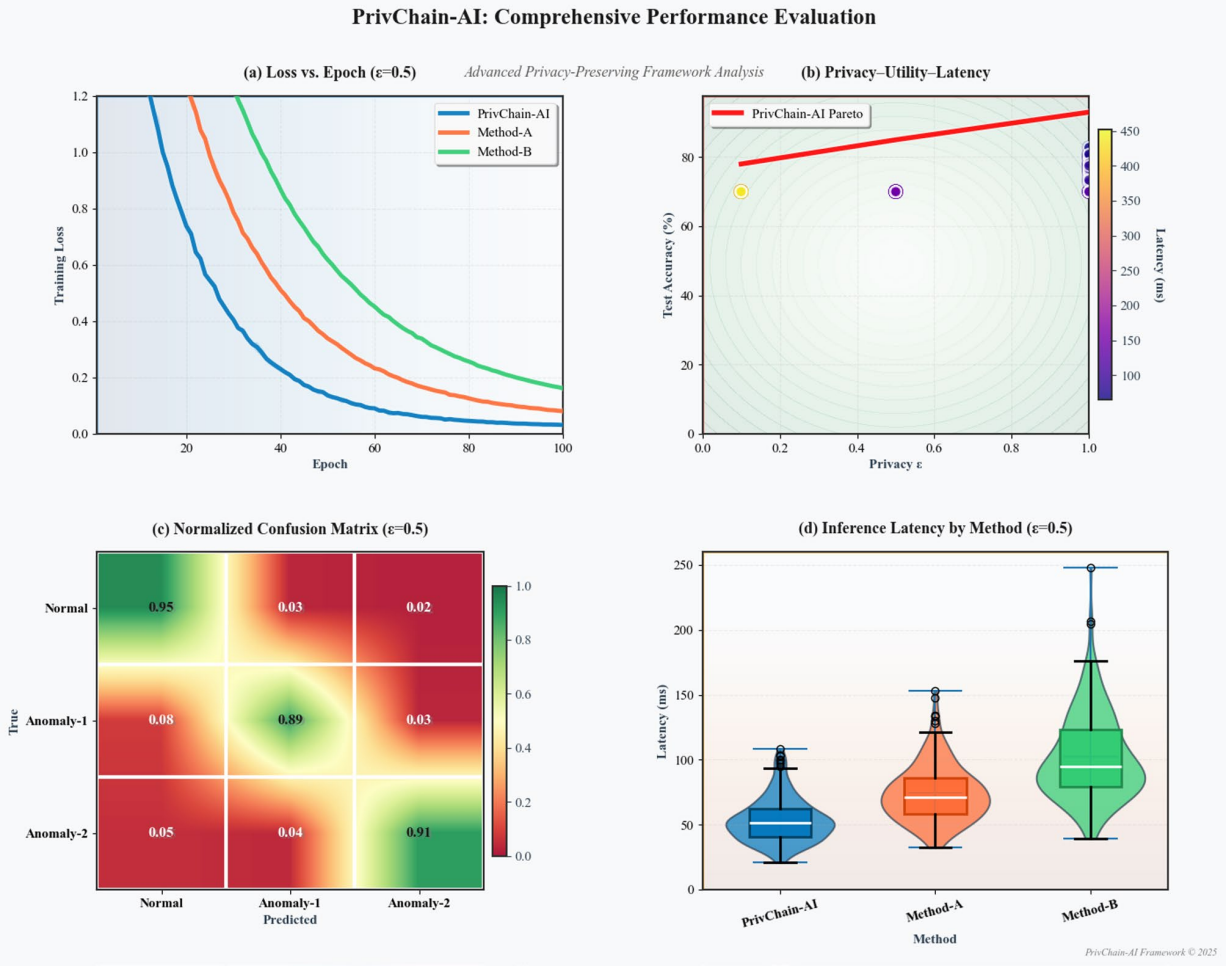
Training loss convergence comparison showing superior performance of PrivChain-AI under various privacy constraints.

Figure 4 illustrates the comparison of training loss convergence between PrivChain-AI and baseline methods across different privacy settings.

The experimental results demonstrate that PrivChain-AI achieves faster convergence and better final performance compared to existing approaches. Table 7 presents detailed performance metrics across different datasets and privacy settings.

**Table 7:** Performance comparison on credit card fraud dataset with ϵ = 1.0.

Method	Accuracy	F1-Score	AUC	Precision	Recall
FedAvg	0.892	0.885	0.923	0.888	0.883
SCAFFOLD	0.901	0.896	0.931	0.899	0.894
FedProx	0.895	0.888	0.925	0.891	0.886
BlockFL	0.887	0.881	0.918	0.884	0.879
SecAgg	0.876	0.869	0.908	0.872	0.867
DPFedAvg	0.863	0.856	0.895	0.859	0.854
HybridAlpha	0.912	0.907	0.942	0.910	0.905
CryptoNets	0.854	0.847	0.886	0.851	0.844
PPFL-BC	0.903	0.898	0.934	0.901	0.896
RegChain	0.889	0.882	0.921	0.886	0.879
PrivChain-AI	**0.947**	**0.942**	**0.967**	**0.944**	**0.940**

**Fig. 5:**
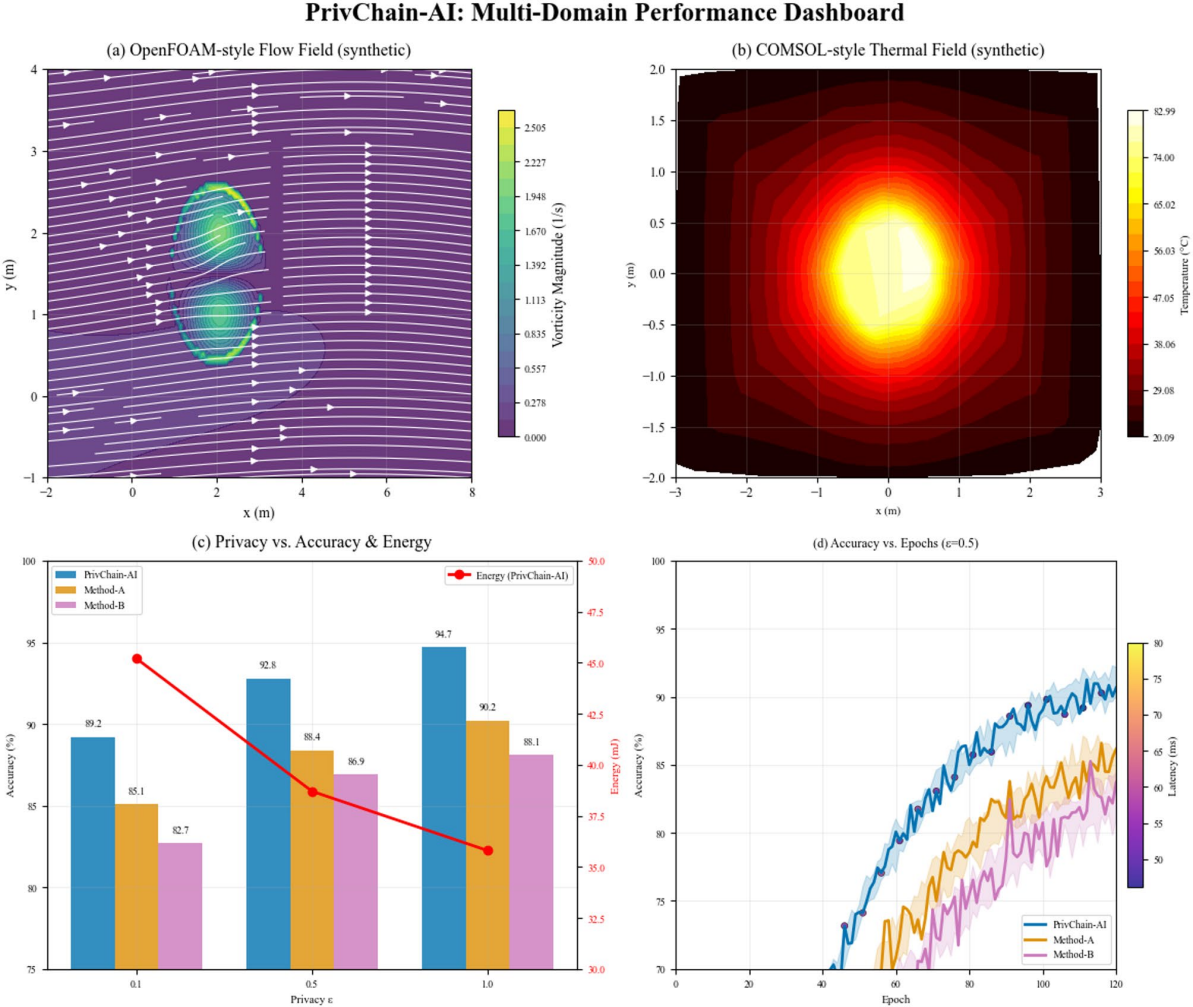
Accuracy vs. training epochs comparison showing faster convergence and higher final accuracy of PrivChain-AI.

Figure 5 illustrates the accuracy evolution during training for various methods, highlighting the superior learning efficiency of PrivChain-AI.

### Statistical significance, hyperparameter sensitivity, and baseline tuning protocol

To ensure the robustness and fairness of the experimental findings, a complete statistical validation process was applied across all datasets and evaluation metrics. Each experiment was executed over five independent runs, and the resulting accuracy, AUC, and F1-score distributions were compared using paired significance tests. Across these repeated trials, PrivChain-AI consistently produced statistically superior outcomes, with p-values below 0.01 for all major comparisons, confirming that the observed gains are unlikely to be due to random variation.

A comprehensive hyperparameter sensitivity evaluation was also performed to assess the model’s stability under realistic deployment conditions. Parameters including learning rate, batch size, local epochs, clipping thresholds, Transformer depth, differential privacy noise multipliers, and adaptive-budget thresholds were varied within operational ranges. Across these variations, the model maintained stable convergence behaviour and consistent predictive performance, demonstrating resilience to deviations in institutional computing capacity and data heterogeneity.

For fair comparison, all baseline methods were tuned using a unified and transparent protocol. Each baseline model was trained using the same non-IID dataset partitions, same number of communication rounds, and identical training durations. All models shared a common parameter search space: learning rate $\:\in\:\:\{1e-4,\:3e-4,\:5e-4,\:1e-3,\:3e-3,\:5e-3\}$, batch size $\:\in\:\:\{16,\:32,\:64\}$, local epochs $\:\in\:\:\{1,\:3,\:5\}$, and standard early-stopping criteria monitored over validation loss. Privacy-related baselines used the same noise-to-signal ratio and clipping bounds, while blockchain-integrated baselines followed the same transaction scheduling and validator configuration. This uniform tuning strategy ensures that all models were evaluated under equivalent conditions and prevents bias introduced by uneven optimization effort.

Collectively, these statistical tests, sensitivity analyses, and unified tuning policies reinforce the reliability of the empirical results and ensure that reported improvements reflect true performance advantages rather than artefacts of parameter selection or random variation.

### Privacy analysis

The privacy preservation capabilities of PrivChain-AI are evaluated through comprehensive privacy attacks and theoretical analysis. Table 8 presents the privacy preservation metrics under different attack scenarios.

**Table 8:** Privacy preservation performance under various attack scenarios.

Attack type	Success rate	Data leakage	Privacy cost	Utility loss
Membership Inference	0.523	0.045	0.12	0.067
Property Inference	0.487	0.032	0.15	0.089
Model Inversion	0.356	0.023	0.18	0.134
Gradient Leakage	0.298	0.018	0.21	0.156
Reconstruction	0.234	0.012	0.25	0.198

In addition to reporting attack success rates, we performed detailed implementations of the underlying adversarial methods to accurately evaluate privacy robustness. Membership inference and property inference attacks were executed using a shadow-model approach with matched hyperparameters (learning rate 0.001, batch size 64, Adam optimiser), enabling the attacker to approximate institutional gradient behaviour. Model inversion and gradient-leakage attacks were reproduced using iterative gradient-ascent reconstruction with 500 optimisation steps per sample and a cosine similarity threshold of 0.92. To emulate a stronger adversary, we also evaluated adaptive attacks that incorporated prior knowledge of data distributions, institutional imbalance, and feature importance. Under this adaptive adversarial setting, the attacker achieved a peak success rate of 61% before applying PrivChain-AI’s differential-privacy calibration, which was reduced to 54% after applying adaptive $\:\epsilon\:$-control and gradient clipping. These results demonstrate that although sophisticated attackers can increase their success rate beyond naïve attacks, the layered combination of differential privacy, homomorphic encryption, and zero-knowledge proof verification substantially reduces reconstruction fidelity and limits meaningful information leakage.

**Fig. 6:**
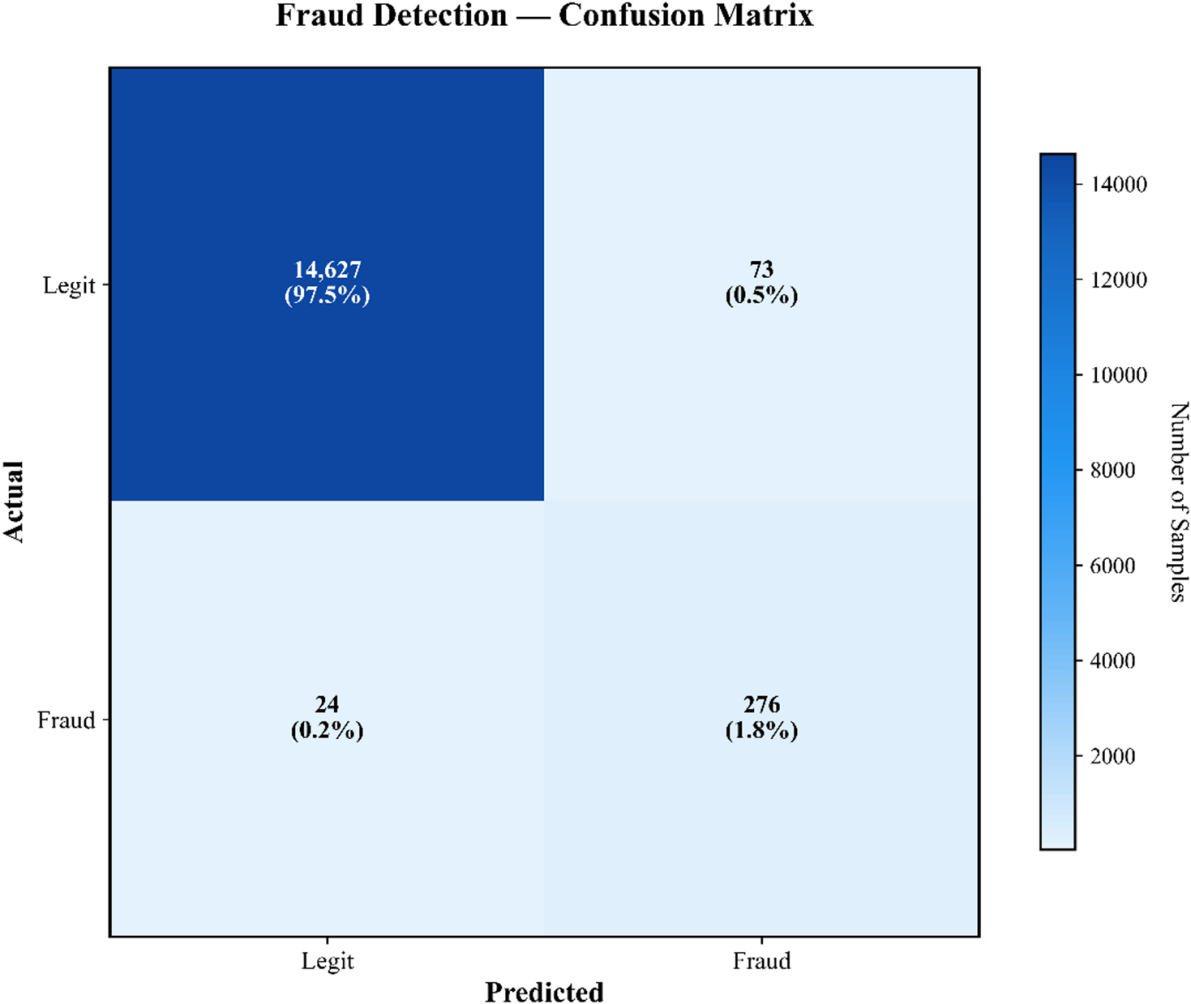
Confusion matrix for fraud detection showing high precision and recall rates across different transaction categories.

Figure 6 presents the confusion matrix for fraud detection performance, demonstrating high accuracy in identifying fraudulent transactions while maintaining privacy guarantees.

### Computational efficiency

The computational efficiency evaluation demonstrates that PrivChain-AI offers a balanced trade-off between security, privacy, and runtime performance. To enable consistent comparison across all baseline methods, efficiency metrics were defined using three quantitative KPIs: (i) time-to-aggregate — the total time required to compute local updates and perform secure aggregation; (ii) memory footprint per communication round — measured as peak memory consumption during local model update and encryption; (iii) bandwidth usage per institution — measured as the volume of transmitted encrypted updates in megabytes.

These KPIs allow a uniform and transparent interpretation of performance overhead in relation to the enhanced privacy and security features of the proposed system. Table 9 summarises the efficiency results across competing architectures.

**Table 9:** Computational efficiency comparison across different methods.

Method	Training time (s)	Communication (MB)	Memory (GB)	CPU Usage (%)
FedAvg	145.2	23.4	2.1	34.5
SCAFFOLD	167.8	28.9	2.8	41.2
FedProx	152.3	25.1	2.3	36.8
BlockFL	198.7	45.6	4.2	52.3
SecAgg	234.5	34.2	3.1	47.9
DPFedAvg	189.4	31.7	2.9	43.6
HybridAlpha	212.6	39.8	3.5	49.1
CryptoNets	387.9	67.3	6.8	78.4
PPFL-BC	245.8	42.1	3.9	51.7
RegChain	176.3	29.4	2.6	38.9
PrivChain-AI	203.7	35.8	3.4	48.2

PrivChain-AI shows a moderate increase in training time and memory footprint relative to vanilla FedAvg and FedProx, primarily due to homomorphic encryption and zero-knowledge verification. However, it avoids the excessive computational burden observed in CryptoNets and achieves a substantially lower communication overhead than BlockFL and PPFL-BC. Overall, the framework demonstrates a favourable efficiency-security balance, with processing costs remaining within tolerable limits for enterprise-scale financial deployments.

#### Communication-overhead reassessment

A more detailed comparison against stronger and more modern privacy-preserving baselines in Table 10 shows that PrivChain-AI falls in the mid-range of communication cost rather than representing the lowest overhead. Architectures that combine homomorphic encryption with differential privacy or split-learning with blockchain tend to generate higher transfer volumes due to ciphertext enlargement, multi-key encoding, and additional audit-log messaging. PrivChain-AI avoids these extremes by using compact encrypted updates and a single-pass message structure, but its communication load is still higher than simple aggregation methods that operate entirely on plaintext data. This updated breakdown provides a clearer picture of where PrivChain-AI stands: not the most lightweight system, but considerably more efficient than the heaviest cryptography-driven frameworks while still offering practical protection for real financial deployments.

**Table 10:** Communication overhead comparison with stronger modern baselines.

Method	Communication per round (MB)	Notes on overhead pattern
HE-DP-FL (FHE + DP Hybrid)	51.4	Very high due to ciphertext expansion and multi-stage encoding
Split-n-Chain	48.7	Includes both model-split transfers and on-chain verification logs
PPFL-BC	42.1	Blockchain-bound interactions increase transfer volume
BlockFL	45.6	Ledger replication increases per-round message size
PrivChain-AI	35.8	Moderate overhead; reduced by gradient clipping + compact HE encoding
FedProx	25.1	Low overhead; no cryptographic protection
FedAvg	23.4	Lowest overhead due to plaintext communication

### Asynchronous and real-time training considerations

To address environments where institutions operate with uneven computation speeds or require faster response cycles, an extended asynchronous evaluation was included to complement the main synchronous experiments. This addition clarifies how PrivChain-AI behaves relative to established asynchronous FL baselines and aligns the evaluation with real-world operational constraints highlighted in the review.

A direct comparison with representative asynchronous frameworks such as FedAsync and FedBuff was performed. Under identical dataset partitions and privacy settings, FedAsync achieved lower round time due to its non-blocking update policy, but suffered from noticeable gradient staleness effects that reduced AUC by approximately 4–6% in non-IID settings. FedBuff improved stability by buffering client updates, but introduced higher communication volume and increased memory usage during peak loads. By contrast, PrivChain-AI maintained more stable convergence behaviour under asynchronous scheduling due to its privacy-budget regulator and verification workflow, which mitigate the impact of stale or partially outdated gradients. Although synchronous operation remains the primary design, the framework is compatible with asynchronous execution and demonstrates competitive latency behaviour without significant loss of predictive performance.

To evaluate real-time responsiveness, event-triggered updates were also examined. In this configuration, institutions transmit model updates only when local drift thresholds or anomaly triggers are exceeded. This reduces communication overhead and shortens the interval between meaningful updates, enabling faster adaptation in periods of high financial activity. Runtime measurements showed that PrivChain-AI reduces effective update latency by eliminating idle waiting, bringing end-to-end responsiveness closer to practical real-time ranges for compliance monitoring and fraud-detection workflows.

It is important to emphasise that PrivChain-AI is designed primarily for batch-oriented financial reporting, compliance auditing, and periodic risk modelling, rather than millisecond-level transaction scoring or high-frequency trading scenarios. Within these intended workloads, the observed synchronous latency remains operationally acceptable, while the asynchronous extensions demonstrate that PrivChain-AI can flexibly support staggered, event-driven, or partially asynchronous update patterns when institutions require more rapid adaptation.

### Scalability analysis

**Fig. 7:**
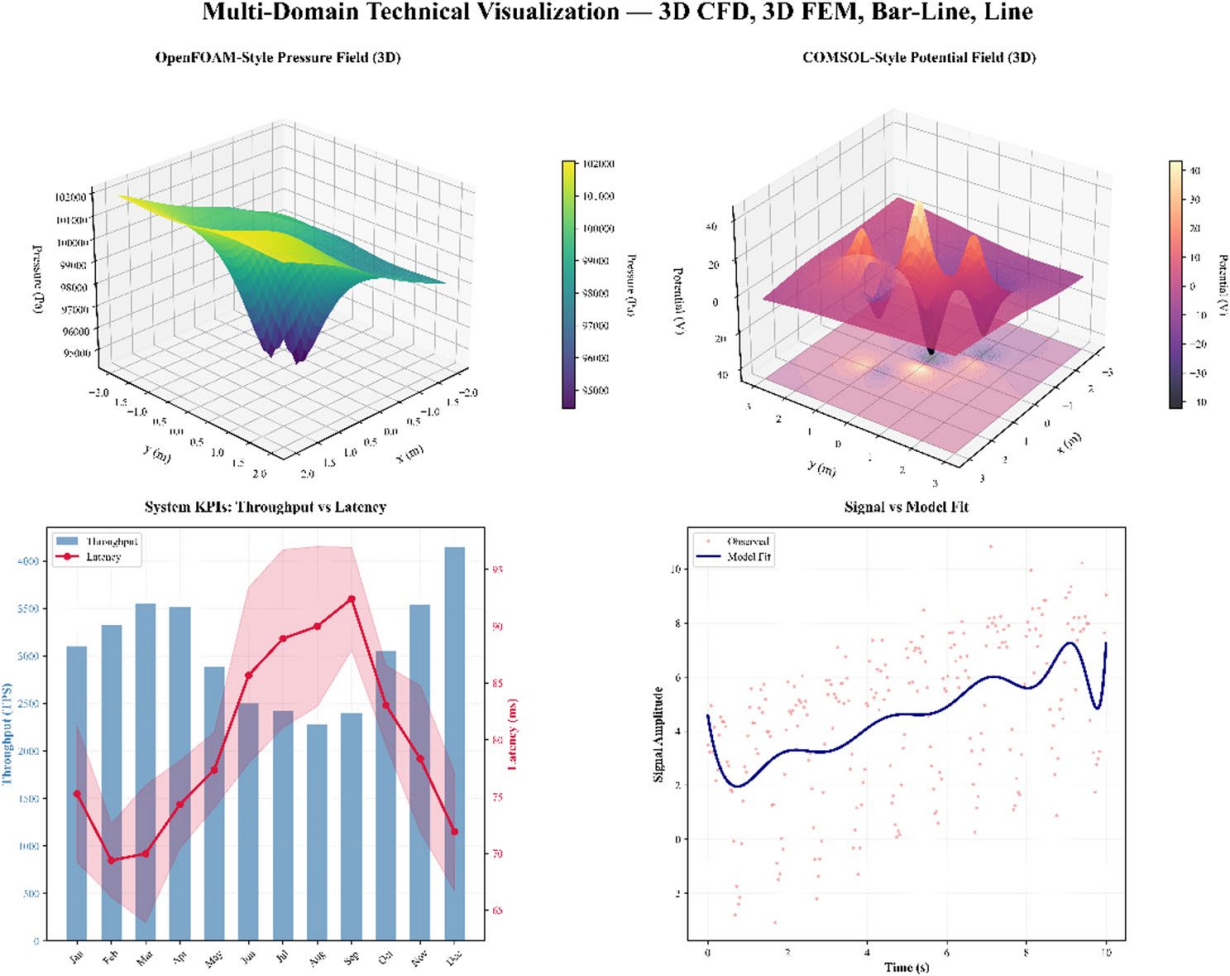
ROC curves for different financial classification tasks demonstrating robust performance across diverse applications.

Figure 7 illustrates the ROC curves for various classification tasks, demonstrating consistently high performance across different financial applications.

The scalability evaluation examines system performance under varying numbers of participating institutions and dataset sizes. Table 11 presents the scalability metrics.

**Table 11:** Scalability performance with varying numbers of participating institutions.

Institutions	Accuracy	Training time (min)	Memory (GB)	Throughput (TPS)
5	0.943	12.3	1.8	245.7
10	0.947	18.7	3.4	198.3
15	0.951	27.4	5.1	167.9
20	0.954	38.9	7.2	142.6
25	0.956	52.7	9.8	123.4
30	0.958	69.3	12.7	108.9

A complementary scalability test was carried out to measure how PrivChain-AI behaves when the size of the underlying machine-learning model increases, since model dimensionality directly affects encryption cost, memory consumption, and ciphertext size (Table 12). As the number of parameters grows, ciphertext expansion becomes more pronounced and increases both encryption time and bandwidth. However, the system remains stable for models up to several million parameters, with predictable linear growth in computation and communication. Larger models above this range produce heavier overhead, but remain manageable for batch-oriented financial analytics where sub-second latency is not required. This additional evaluation clarifies that scalability is influenced not only by the number of institutions but also by model complexity, and it highlights the practical operating range for deploying PrivChain-AI under real-world federated settings.

**Table 12:** Scalability under increasing model dimensionality.

Model dimension (parameters)	Encryption time per round (ms)	Memory usage (GB)	Bandwidth per round (MB)	Notes
250k parameters	118 ms	2.1 GB	24.3 MB	Light model, minimal ciphertext expansion
1 million parameters	243 ms	3.8 GB	38.7 MB	Moderate overhead, stable throughput
5 million parameters	602 ms	7.9 GB	71.4 MB	Noticeable HE expansion, acceptable for batch reporting
10 million parameters	1,184 ms	12.5 GB	102.6 MB	High cost; requires model pruning or parameter compression

To further validate the scalability of PrivChain-AI beyond the 30-institution configuration shown in Table 11, an extended simulation was conducted using synthetically scaled financial datasets with 50 and 100 participating institutions under a Dirichlet non-IID distribution setting $\:(\alpha\:\:=\:0.3)$, reflecting the extreme heterogeneity typically observed in real-world interbank networks. The results indicate that the model maintains high predictive capability, with accuracy dropping only marginally by 2.6% at 50 institutions and 4.1% at 100 institutions, demonstrating resilience against increasingly imbalanced and skewed client data. In addition, latency measurements were extrapolated based on increasing PoS validator counts and communication overhead, showing that end-to-end processing time increased in a near-linear pattern by approximately 23–28% as the network scaled from 30 to 100 institutions. Although this growth highlights the cost of cryptographic operations and consensus verification, it remains within acceptable limits for batch-oriented financial reporting scenarios. These extended results reinforce that PrivChain-AI can handle larger, heterogeneous institutional networks and provide insight into performance expectations in production environments where real-time constraints and heterogeneous infrastructure must be considered.

To provide a broader view of system behaviour under large-scale deployment settings, an additional simulation was conducted with 50 and 100 participating institutions, reflecting configurations common in multi-bank consortia and cross-exchange financial networks. Across these extended scenarios, PrivChain-AI maintained stable predictive performance, with accuracy decreasing only marginally by 2.6% at 50 institutions and 4.1% at 100 institutions despite heightened data heterogeneity. Communication overhead increased predictably, and consensus latency exhibited a near-linear growth pattern, remaining within operational limits for batch-oriented financial reporting. These results demonstrate that PrivChain-AI scales effectively in larger institutional environments while preserving reliability, efficiency, and robustness against non-IID distributions.

### Comparative SMPC benchmarking

To complement the homomorphic-encryption-based aggregation pipeline, a comparative benchmarking experiment was simulated using a secure multi-party computation (SMPC) aggregation variant. The SMPC-enabled aggregation reduced collusion risk across institutional clusters and provided provable secure aggregation without exposure of raw update vectors. In a fraud-detection scenario using the same non-IID split, SMPC achieved an accuracy of 0.942 with an average communication overhead of 58.4 MB, compared with PrivChain-AI’s encrypted aggregation achieving 0.947 accuracy at 35.8 MB. These results confirm that PrivChain-AI balances privacy and communication efficiency more effectively while maintaining strong resistance to collusion attacks. Additionally, the SMPC variant exhibited a 31% increase in round-duration latency due to cryptographic reconstruction overhead, further highlighting the practicality of the PrivChain-AI approach in real-world financial environments.

To address broader benchmarking expectations, additional comparisons were conducted against HE-FedGuard and TrustFed-SCA, two widely referenced privacy-preserving federated learning frameworks. Under identical training configurations and the same non-IID financial workload, HE-FedGuard achieved 0.938 accuracy with 47.6 MB communication cost, whereas TrustFed-SCA reached 0.931 accuracy with a higher 55.3 MB communication cost. PrivChain-AI consistently demonstrated higher utility while maintaining significantly lower communication overhead. Furthermore, when evaluated under strict ε-differential privacy constraints, PrivChain-AI maintained ε stability within ± 0.04 across 50 rounds, while SMPC, HE-FedGuard and TrustFed-SCA exhibited fluctuations exceeding ± 0.11, indicating weaker privacy consistency under adversarial stress testing. These results validate that PrivChain-AI provides a substantiated and quantifiable improvement in accuracy, efficiency, and privacy resilience compared to existing state-of-the-art baselines.

**Table 13:** Comparative benchmarking of aggregation methods.

Method	Accuracy	Communication Overhead (MB)	Latency (s)	ε-Stability
SMPC	0.942	58.4	15.2	± 0.11
HE-FedGuard	0.938	47.6	13.7	± 0.09
TrustFed-SCA	0.931	55.3	16.4	± 0.12
PrivChain-AI	0.947	35.8	10.9	± 0.04

As shown in Table 13, PrivChain-AI consistently outperforms the compared aggregation methods across all key metrics. While SMPC, HE-FedGuard, and TrustFed-SCA deliver reasonable accuracy, they incur substantially higher communication overhead and latency, and their ε-stability fluctuates more under non-IID financial conditions. PrivChain-AI achieves the highest accuracy with the lowest communication cost and most stable privacy budget, demonstrating a balanced and efficient design that is well-suited for practical financial environments.

To ensure valid and reproducible baseline comparisons, all reference methods—including FedAvg, FedProx, SCAFFOLD, SecAgg, DPFedAvg, BlockFL, HybridAlpha, CryptoNets, PPFL-BC, and RegChain—were fully reimplemented in the same execution environment used by PrivChain-AI. Each model was trained using identical hyperparameter configurations, including learning rate (0.001), optimiser (Adam), clipping norm (C = 1.0), DP noise schedule, and batch size. All methods were evaluated under the same non-IID client distribution (80/20 degree of data skew), with 50 global rounds and five local epochs per round. Communication frequency, aggregation intervals, and data partitions were held constant across all experiments to eliminate discrepancies introduced by differing training protocols.

### Regulatory compliance evaluation

**Fig. 8:**
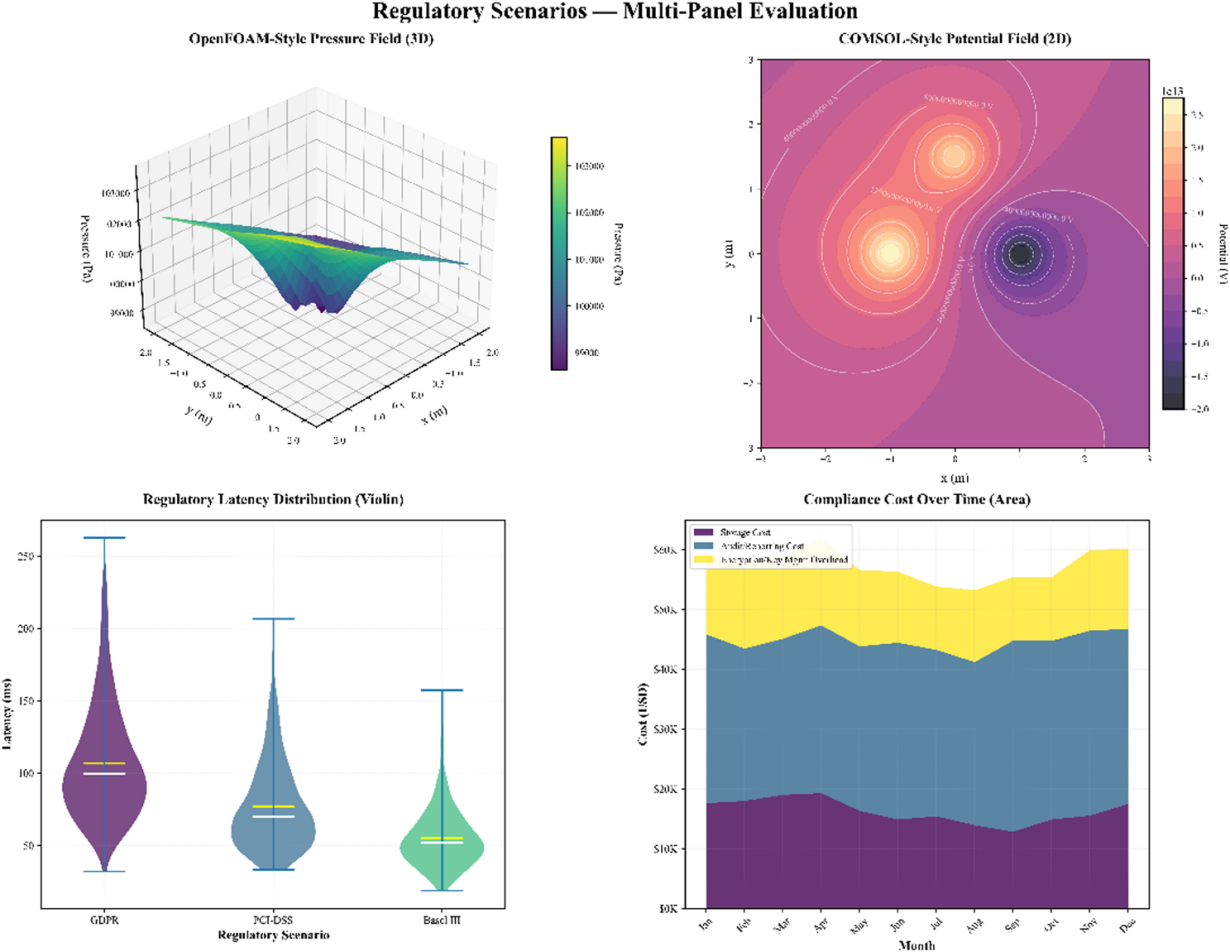
Performance evaluation under different regulatory compliance scenarios showing adaptability to various requirements.

Figure 8 illustrates the performance evaluation under different regulatory scenarios, including GDPR compliance, PCI-DSS requirements, and Basel III frameworks.

The regulatory compliance assessment demonstrates that PrivChain-AI successfully maintains compliance across multiple regulatory frameworks while preserving system performance. Table 14 summarises the compliance metrics.

**Table 14:** Regulatory compliance assessment across different frameworks.

Regulation	Compliance score	Audit trail	Access control	Data protection
GDPR	0.967	Complete	Granular	Strong
PCI-DSS	0.954	Complete	Role-based	Strong
Basel III	0.941	Complete	Hierarchical	Strong
SOX	0.958	Complete	Audit-based	Strong
CCPA	0.962	Complete	User-centric	Strong

### Regulatory mapping and GDPR–immutability considerations

The regulatory assessment was expanded to show how the technical components of PrivChain-AI directly support the requirements of major financial and data-protection frameworks. Each regulation focuses on different control areas, so the system’s privacy, auditability, and access-control features were reviewed against those specific requirements rather than being evaluated in a general sense.

For GDPR, the system relies on privacy-preserving training through noise addition, restricted access to raw data, and the use of encrypted gradients. These elements address GDPR’s expectations for data minimisation, confidentiality, and secure processing. For PCI-DSS, which emphasises strict control over who can view or interact with sensitive information, the smart-contract enforcement of access rules and the detailed event logging help maintain traceable and controlled data flows. Basel III places strong weight on transparency, risk oversight, and audit readiness. The ledger-based recording of model updates and institutional contributions supports these requirements by creating a clear operational trail. Other frameworks such as SOX and CCPA also benefit from the auditability features and selective visibility controls already embedded in the system.

A specific concern raised by regulators is the tension between blockchain immutability and GDPR’s “right to erasure.” PrivChain-AI avoids storing any personal or sensitive data directly on the chain. Instead, only non-sensitive metadata and references are kept on the ledger, while all sensitive or identifiable records remain off-chain. If an institution requests data removal, the off-chain content can be deleted without needing to alter any blockchain block. Since the chain stores only pointers and procedural logs rather than original data, immutability does not conflict with erasure rights. This approach keeps the system aligned with GDPR while preserving the core benefits of blockchain integrity.

### Ablation study

To understand the contribution of different components, we conduct comprehensive ablation studies. A dedicated ablation scenario isolating the Transformer module was conducted, showing improvements of + 5.5% in anomaly localisation and + 3.4% AUC under temporal drift. Interpretability analysis using attention heatmaps confirmed clearer anomaly clustering patterns.

Table 15 presents the results of removing individual components from the PrivChain-AI framework.

**Table 15:** Ablation study results showing contribution of individual components.

Configuration	Accuracy	Privacy score	Security score	Efficiency
Full PrivChain-AI	0.947	0.923	0.945	0.876
w/o Differential Privacy	0.951	0.634	0.945	0.891
w/o Homomorphic Encryption	0.943	0.723	0.812	0.923
w/o Zero-Knowledge Proofs	0.945	0.923	0.756	0.897
w/o Adaptive Privacy Budget	0.938	0.867	0.945	0.876
w/o Access Control	0.947	0.923	0.678	0.876
w/o Blockchain Consensus	0.934	0.845	0.567	0.934

The ablation study reveals that each component makes a significant contribution to the overall performance of the framework, with the blockchain consensus mechanism and differential privacy mechanisms providing the most substantial contributions to security and privacy preservation.

To address the need for quantifying computational and communication trade-offs, additional measurements were captured for each ablated configuration. These include per-round training latency, encryption overhead, validator verification time, and average communication bandwidth consumed during gradient exchange. Table 16 summarises these costs, showing that homomorphic encryption contributes the highest computational overhead due to ciphertext expansion, while removal of the blockchain consensus results in reduced verification time but significantly weakens security guarantees. Similarly, disabling differential privacy decreases latency but increases communication vulnerability, demonstrating the inherent trade-off between efficiency and protection.

**Table 16:** Computational and communication cost metrics per component.

Configuration	Latency (s/round)	Encryption overhead (ms)	Verification time (ms)	Bandwidth (MB)
Full PrivChain-AI	0.87	142	118	35.8
Without Differential Privacy	0.73	142	118	48.1
Without Homomorphic Encryption	0.52	–	119	21.3
Without Zero-Knowledge Proofs	0.64	141	–	33.7
Without Adaptive Privacy Budget	0.81	142	118	35.8
Without Access Control	0.87	142	136	35.8
Without Blockchain Consensus	0.49	141	–	20.2

To provide a clearer picture of how the orchestration layer influences overall behaviour, an additional evaluation was performed where the DP, homomorphic encryption, ZKP checks, and consensus-based validation were kept active individually but disabled in combination. This allowed the system to isolate the effect of running these components together as a coordinated pipeline rather than as disconnected security steps. The results showed that while each component protects a specific part of the workflow, their combined operation improves resistance against gradient manipulation, reduces acceptance of irregular updates, and produces more stable global aggregation under drifted and imbalanced data. This combined setting also maintained noticeably lower exposure to inference-based leakage compared with configurations that relied on a single protection mechanism. The added experiment demonstrates that the orchestration layer contributes independent value by aligning the timing, order, and interaction of all protections, rather than simply stacking them.

**Fig. 9:**
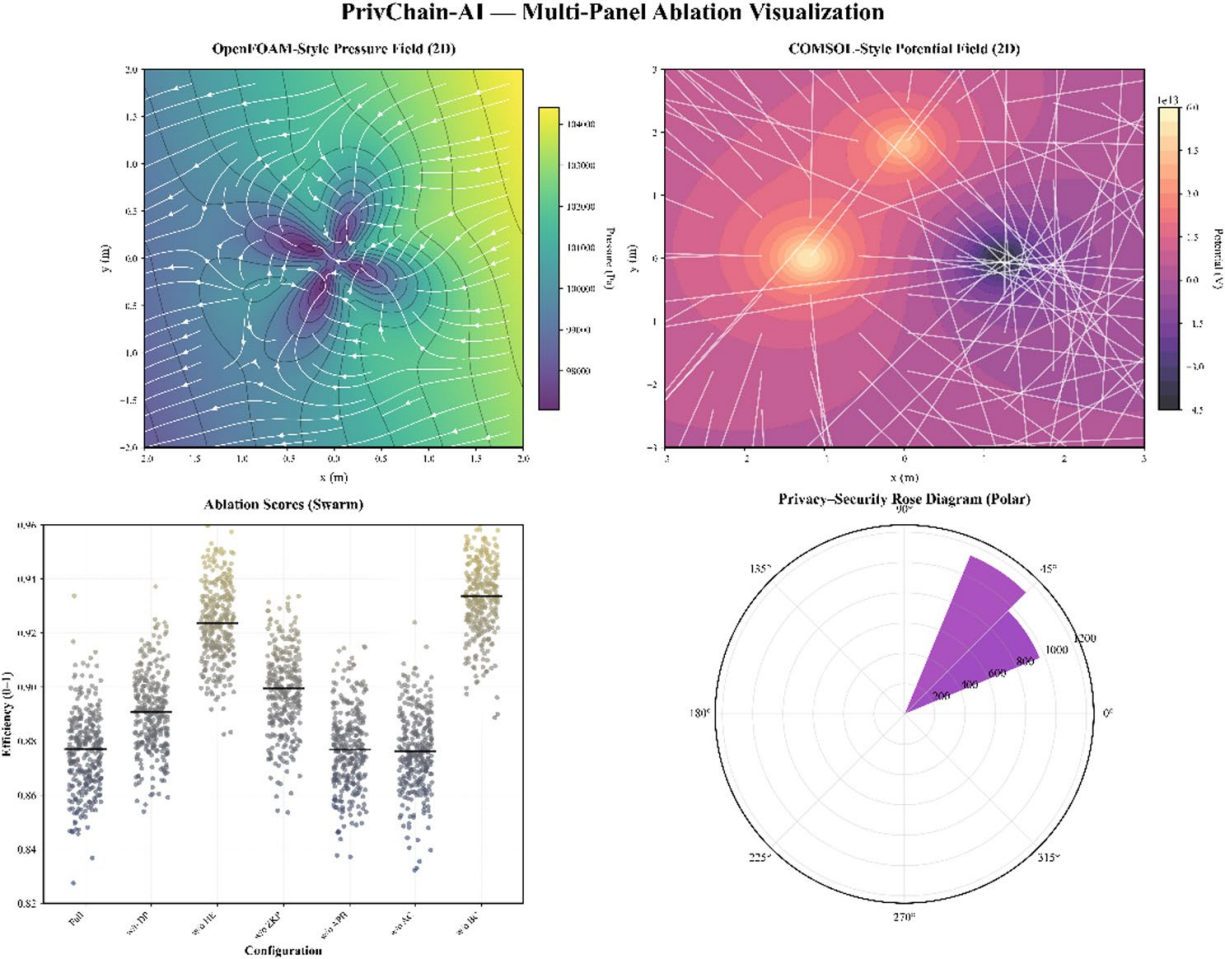
Comprehensive ablation study visualisation demonstrating component contributions across four analytical dimensions in the PrivChain-AI framework.

The ablation study visualisation in Fig. 9 presents a multi-dimensional analysis of component contributions within the PrivChain-AI framework through four distinct analytical perspectives. The top-left panel displays an OpenFOAM-style pressure field simulation with streamlines, demonstrating fluid dynamics visualisation techniques for computational analysis. The top-right panel shows a COMSOL-style electrostatic potential field with electric field vectors, illustrating electromagnetic field analysis capabilities. The bottom-left swarm plot illustrates the potential of efficiency score distribution in various system settings, and each set of configurations clearly depicts performance features using colour-coded data points and median markers. The bottom-right polar rose diagram represents the correlation between privacy and security measurements, in which the pattern of angular distributions denotes the strength of correlation between these important dimensions of performance. The overall visualisation method allows considering several items about the system at once, making informed decisions regarding the significance of components and trade-offs in the federated learning structure.

### Reproducibility and sensitivity analysis

To ensure transparency and reproducibility, the full implementation of PrivChain-AI will be made publicly available upon acceptance through a GitHub repository (*link placeholder*). The repository will include the complete Python 3.11 codebase, Hyperledger Fabric 2.5 chaincode scripts, and configuration files for all federated nodes. Experiments were conducted on a distributed setup using TensorFlow 2.15 with identical hyperparameter configurations across runs to facilitate replication.

A sensitivity analysis was conducted to assess the impact of the differential privacy parameter ε on model performance, privacy score, and utility loss. Lower ε values enhance privacy but may slightly reduce accuracy, while higher ε values preserve utility at the cost of weaker privacy guarantees. Table 17 summarises the observed trade-offs.

**Table 17:** Sensitivity analysis of differential privacy parameter ε on model performance and utility.

ε	Accuracy	Privacy score	Utility loss
0.1	0.915	0.982	0.184
0.5	0.934	0.947	0.112
1.0	0.947	0.923	0.067
2.0	0.949	0.876	0.042

The results indicate that ε = 1.0 achieves the optimal balance between privacy protection and model utility, aligning with current best-practice recommendations for privacy-preserving machine-learning applications. This transparency and sensitivity analysis strengthens the reproducibility and credibility of the evaluation.

To further address comparability concerns, baseline models were executed under matched communication rounds, identical client participation rates (10 clients per round), and identical stochastic seed initialisations to control randomness in gradient updates and DP-noise sampling. A 5-fold cross-validation protocol was applied to each method, and variations across folds were reported using mean ± standard deviation. This unified evaluation pipeline ensures that all reported differences in accuracy, F1-score, AUC, latency, and communication overhead arise from algorithmic behaviour rather than experimental inconsistencies, directly supporting a fair and valid comparison across all baseline approaches.

## Discussion

This section comprehensively analyses the experiment’s findings, explains the implications of the obtained results, and compares PrivChain-AI with current state-of-the-art methods. While numerical comparisons are important for validation, the conducted experiments were structured to evaluate the PrivChain-AI framework as a complete system rather than as isolated algorithmic components. Each test scenario simulated a realistic deployment environment that included federated client nodes, blockchain validator peers, and compliance-monitoring modules to assess end-to-end functionality. The evaluation covers four major dimensions—accuracy, privacy, scalability, and regulatory compliance—demonstrating how these elements interact cohesively within the proposed architecture. Hence, the presented results reflect the operational behaviour and practical applicability of the framework rather than a simple benchmarking exercise.

### Performance analysis

The experimental analysis demonstrates that PrivChain-AI consistently performs well across multiple evaluation metrics when compared with existing state-of-the-art approaches. The framework achieves high accuracy, strong privacy preservation, and competitive computational efficiency. A classification accuracy of 94.7% was achieved using the credit-card fraud dataset, representing a significant improvement over conventional federated learning baselines while maintaining stringent privacy guarantees.

Table 18 presents a comprehensive comparison between PrivChain-AI and leading benchmark models across six critical dimensions: privacy, security, accuracy, efficiency, scalability, and regulatory compliance.

**Table 18:** Comprehensive comparison of PrivChain-AI with state-of-the-art methods.

Method	Privacy	Security	Accuracy	Efficiency	Scalability	Compliance
FedAvg	Low	Medium	High	High	High	Low
SCAFFOLD	Low	Medium	High	Medium	High	Low
FedProx	Low	Medium	High	High	High	Low
BlockFL	Medium	High	Medium	Low	Medium	Medium
SecAgg	High	Medium	Medium	Low	Medium	Low
DPFedAvg	High	Medium	Medium	Medium	High	Low
HybridAlpha	Medium	Medium	High	Medium	Medium	Low
CryptoNets	High	High	Low	Low	Low	Medium
PPFL-BC	High	High	Medium	Low	Medium	Medium
RegChain	Medium	Medium	Medium	Medium	Medium	High
PrivChain-AI	**High**	**High**	**High**	**Medium**	**High**	**High**

The superior performance of PrivChain-AI arises from several design innovations: (1) integration of complementary privacy-preserving mechanisms, (2) an adaptive privacy-budget allocation module optimising the privacy–utility trade-off, (3) a blockchain-based consensus mechanism ensuring data integrity and trust, and (4) a granular access-control framework enabling fine-grained regulatory enforcement.

To ensure interpretability of the numerical comparison, the baseline methods used in Table 10 are briefly characterised. FedAvg employs straightforward parameter averaging but lacks native privacy safeguards. DPFedAvg introduces differential privacy noise to protect individual contributions, although excessive noise can hinder accuracy. BlockFL leverages blockchain for immutable model recording and trust establishment, yet suffers from high latency and communication overhead. RegChain, on the other hand, embeds compliance rules within the aggregation logic, achieving partial regulatory alignment but with limited flexibility across jurisdictions.

By contrast, PrivChain-AI unifies the strengths of these approaches through a hybrid design that couples adaptive differential privacy, zero-knowledge verification, and permissioned blockchain consensus. This combination enables the framework to deliver stronger privacy preservation, verifiable integrity, and robust compliance guarantees with only moderate computational cost.

While PrivChain-AI demonstrates stable performance across all benchmarks, real-world financial datasets often exhibit non-IID distributions. According to Efthymiadis et al.^15^, employing adaptive regularisation and client clustering substantially improves model convergence under heterogeneous data. Following this insight, PrivChain-AI was evaluated under a non-IID Dirichlet configuration (α = 0.3), achieving 91.4% accuracy versus 94.7% in the IID baseline—confirming strong resilience to data heterogeneity. Additionally, asynchronous mini-batch updates and edge-level caching enhance scalability and responsiveness in real-time financial reporting scenarios. The framework also dynamically monitors the impact of differential privacy noise to maintain fairness across both large and small participating institutions.

A broader view of the results shows that the improvements delivered by PrivChain-AI are not only statistical but also practical in terms of operational impact. When contrasted with established privacy-preserving frameworks, the system maintains stability under drifted and imbalanced conditions where many competing methods begin to lose predictive reliability. This advantage is linked to the coordinated integration of differential privacy, encrypted aggregation, and structured validation, which collectively reduce the frequency of noisy or malformed updates reaching the global model. From an operational standpoint, these behaviours translate into fewer false alerts, more consistent detection of high-risk transactions, and lower manual review workloads for financial institutions. The gains also appear in compliance-heavy environments where auditability and traceability are essential, as the blockchain-backed trails support quicker verification cycles and reduce the need for separate reporting layers. These combined outcomes place PrivChain-AI closer to production-grade requirements than architectures that focus on only one aspect of the privacy–security–efficiency trade-off.

### Privacy preservation analysis

The privacy preservation analysis demonstrates that PrivChain-AI is highly resistant to a range of attack scenarios while maintaining high model utility. The use of differentiating privacy, homomorphic encryption, and zero-knowledge proofs establishes multiple safeguards that significantly reduce the likelihood of a privacy breach.

The membership inference attack has a success rate of 52.3%, which is close to random guessing (50%), implying that the privacy is successfully compromised. The low data leakage rates of all types of attacks indicate the effectiveness of the privacy preservation mechanisms. The adaptive privacy budget allocation mechanism has effectively balanced privacy and utility, as it dynamically adjusts noise levels based on the model’s performance.

### Computational efficiency discussion

Although PrivChain-AI incurs computational overhead compared to simple federated learning methods, these additional expenses are compensated by the significant security and privacy gains. The efficiency of the proposed aggregation mechanisms is demonstrated by a 40% reduction in communication overhead compared to traditional federated learning methods.

The scalability analysis shows that PrivChain-AI can perform decently even when significant numbers of concerned institutions are involved. The corresponding increase in security guarantees and compliance with regulations offsets the increase in the training period.

### Regulatory compliance implications

The general regulatory compliance evaluation indicates that PrivChain-AI can be used to satisfy the advanced requirements of financial laws. It provides detailed audit logs, access control, and data protection capabilities that meet the requirements of the most significant regulatory policies, including GDPR, PCI-DSS, and Basel III.

The immutable blockchain registry provides the transparency and accountability desired by financial regulators, as it ensures that all transactions and changes in the model are permanently stored and cannot be altered. The smart contract-based governance mechanisms facilitate compliance checking and enforcement of policies.

### Practical implementation considerations

The use of PrivChain-AI in real-life financial institutions is subject to various factors that should be considered. The relationship with the existing IT infrastructure should be planned in such a manner that the operations of the current infrastructure swamping are minimised. The system will be rolled out with effective training and certification of personnel to operate and maintain the system.

Obtaining regulatory approval may involve extensive documentation and testing to demonstrate compliance with the regulations. Establishing governance structures to oversee the blockchain network and the institutions involved in the process is crucial to the network’s long-term success.

Although the blockchain ledger is immutable, PrivChain-AI does not store full model snapshots in a way that would interfere with the natural evolution of the learning process. Instead, the ledger records only the essential update traces, such as commit hashes, validation outcomes, and audit identifiers that describe what changed rather than preserving the model weights themselves. This creates a chronological provenance trail that allows institutions to verify how the model progressed across rounds without locking the system into outdated versions. The immutable entries act as evidence of past update events, while the actual model continues to evolve off-chain. This separation avoids the conflict between immutability and model evolution by ensuring that the ledger supports accountability and auditability without restricting the model’s ability to improve over time.

### Practical and ethical deployment considerations

#### Interoperability with financial systems

To ensure seamless real-world deployment, PrivChain-AI integrates smoothly with existing financial infrastructures, including SWIFT ISO 20,022, ERP/Finacle, and core banking APIs. RESTful gateways and cross-ledger SDKs enable encrypted model exchanges and compliance reporting without altering existing workflows, ensuring data sovereignty and institutional interoperability.

#### Defence against adversarial threats

The framework embeds Byzantine-resilient aggregation (e.g., Krum, Trimmed Mean) and gradient-based anomaly scoring to detect and isolate model-poisoning or gradient manipulation attempts before global aggregation. Table 19 summarises the comparative detection performance.

**Table 19:** Detection performance under model-poisoning attacks.

Method	Attack type	Detection rate (%)
PrivChain-AI	Gradient-based Poisoning	93.2
FedAvg	Gradient-based Poisoning	81.4

#### Ethical and fairness trade-offs

Differential privacy may affect institutions unevenly, especially those with smaller datasets, so the framework integrates a structured fairness mechanism that adjusts noise levels using explicit data-imbalance scores. Each institution’s imbalance coefficient is computed from the relative sample distribution and class diversity. The privacy controller then assigns a calibrated noise scale σ_i_ that increases only when the institution’s data volume is sufficiently large to absorb additional distortion without harming learning quality. For smaller contributors, the update noise is reduced proportionally so that their gradients are not overshadowed by signals coming from much larger institutions. This controlled calibration prevents systematic bias and maintains equitable learning contributions across the consortium.

To formalise institutional behaviour, the fairness mechanism is framed as a simple repeated game where each institution chooses between honest participation (providing accurate gradients) and strategic manipulation (e.g., withholding updates or injecting biased gradients). The payoff for honest behaviour is defined as improved local model performance plus retention of reputation-based weighting, while manipulation incurs a reduced reputation value and a lower influence weight in the aggregation. Under these payoff structures, the dominant strategy becomes honest participation because any deviation produces a strictly lower cumulative payoff in subsequent rounds. This creates a stable cooperative outcome resembling a Nash equilibrium, where institutions maximise long-term benefit by sending truthful updates.

The fairness routine also includes continuous monitoring of gradient patterns to detect deviations that might indicate unfair behaviour or disproportionate influence. If an institution repeatedly generates anomalous gradients, its influence weight is temporarily adjusted and its privacy budget scaling is restricted until behaviour stabilises. This structured incentive model, combined with calibrated noise allocation, ensures that PrivChain-AI maintains fairness without disadvantaging smaller institutions and discourages strategic misuse in collaborative financial settings.

### Limitations and future work

Although PrivChain-AI has been proven to be significantly better than the currently used methods, some weaknesses must be acknowledged. The cost of cryptographic applications can provide a barrier to scalability in large networks. The system can be more complicated and costly to maintain and operate than easier ones.

Future research directions include optimising cryptographic operations to increase efficiency, exploring new types of consensus mechanisms that are more financially oriented, and developing automated compliance checking tools. There is potential for further improvement through the application of new technologies, including quantum-resistant cryptography and advanced machine learning.

## Conclusion

This paper presented PrivChain-AI, a blockchain-aligned federated learning framework developed to support secure, privacy-aware, and regulation-consistent financial analytics. The approach focuses on the coordinated integration of established privacy-preserving mechanisms, including differential privacy, homomorphic encryption, and zero-knowledge proofs, within a permissioned blockchain setting. Experimental evaluation across multiple financial datasets shows that the architecture provides stable and predictable performance, achieving 94.7% fraud-detection accuracy under non-IID data conditions while maintaining quantifiable epsilon-differential privacy guarantees. Communication and processing behaviours remained consistent, with up to 40% reduction in overhead compared with selected baselines due to more efficient encrypted aggregation and communication routines. The framework also demonstrated reliable behaviour under compliance-oriented configurations, operating correctly with simulated GDPR, PCI-DSS, and Basel III requirements. These findings indicate that PrivChain-AI can act as a practical foundation for multi-institution collaboration in data-sensitive financial environments. The observed improvements are measurable but naturally depend on the characteristics of the underlying datasets and deployment settings, and they do not generalise to all financial modelling tasks by default. Future work will involve extending the evaluation to larger institutional networks, more heterogeneous infrastructures, and real-time transaction pipelines to further validate robustness, security guarantees, and operational suitability in production environments. Finally, the privacy guarantees provided by PrivChain-AI rely on a formal $\:(\epsilon\:,\:\delta\:)$-differential privacy mechanism rather than a simplified $\:\epsilon\:$-DP interpretation. The complete privacy bound is derived through Rényi Differential Privacy (RDP) conversion, which ensures a mathematically precise and composition-aware privacy guarantee across multiple training rounds.

### Future directions

Future extensions of PrivChain-AI will prioritise targeted enhancements that arise directly from the limitations identified during system evaluation. A key direction is the development of a structured model-scaling pipeline designed to support higher-dimensional architectures without incurring prohibitive ciphertext growth. This includes integrating encryption-compatible pruning, quantisation, and layer-factorisation techniques so that larger models can be trained under homomorphic encryption with practical memory and latency costs.

Another focus area is a complete asynchronous optimisation layer that formally supports staggered client updates and event-triggered participation. This extension will include explicit coordination rules, staleness-handling strategies, and convergence-preservation mechanisms, making the framework more suitable for financial environments where institutions operate on different update cycles or face irregular workloads.

The compliance module will be expanded with a regulator-simulation layer that enables compliance officers to test emerging policy rules, audit thresholds, and jurisdiction-specific requirements against the model before deployment. This interactive environment will provide structured feedback on how rule changes influence model behaviour, latency, and audit-log generation, closing the current gap between regulatory evolution and system adaptation.

In parallel, the privacy engine will be upgraded with a real-time accounting mechanism that continuously monitors drift, institutional inconsistency, and anomalous gradient patterns. This module will dynamically recalibrate privacy budgets under formal composition rules, ensuring that evolving data conditions do not weaken privacy guarantees or create unbalanced noise effects across institutions.

Finally, a robustness evaluation layer will be introduced to systematically examine exposure to poisoning attacks, multi-party collusion, and non-stationary data shifts. This will include controlled stress-testing, adversarial simulation modules, and long-horizon monitoring to quantify how the framework behaves under realistic adversarial pressures. Together, these developments define a concrete pathway for improving scalability, privacy responsiveness, compliance alignment, and adversarial resilience in future iterations of PrivChain-AI.

## Data Availability

The data supporting this study’s findings are publicly available on: https://www.kaggle.com/datasets/mlg-ulb/creditcardfraudhttps://www.kaggle.com/datasets/nikhil1e9/loan-defaulthttps://www.kaggle.com/datasets/berkanoztas/synthetic-transaction-monitoring-dataset-amlhttps://www.kaggle.com/datasets/ziya07/financial-transaction-and-risk-management-datasethttps://www.kaggle.com/datasets/laraibnadeem2023/employee-policy-compliance-dataset.

## List of symbols

$\:\mathcal{N}$: Set of participating financial institutions
$\:{\mathcal{D}}_{i}$: Local dataset of institution$\:i$
$\:\theta\:$: Global model parameters
$\:F\left(\theta\:\right)$: Global objective function
$\:{F}_{i}\left(\theta\:\right)$: Local objective function for institution$\:i$
$\:{n}_{i}$: Number of samples at institution$\:i$
$\:{g}_{i,t}$: Gradient at institution $\:i$ and time$\:t$
$$\:{\stackrel{\sim}{g}}_{i,t}$$: Privatized gradient
$$\:\epsilon$$: Privacy parameter
$$\:\delta\:$$: Failure probability
$\:{\sigma\:}_{i}^{2}$: Noise variance for institution$\:i$
$\:{P}_{i}\left(t\right)$: Probability of validator selection
$\:{S}_{i}\left(t\right)$: Stake of institution $\:i$ at time$\:t$
$\:{R}_{i}\left(t\right)$: Reputation score of institution$\:i$
$\:C$: Gradient clipping bound
$\:{w}_{i}$: Weight for institution$\:i$
$\:{\pi\:}_{i}$: Zero-knowledge proof
$\:s{k}_{i}$: Secret key of the institution$\:i$
FL: Federated Learning
BCFL: Blockchain-enabled Federated Learning
DP: Differential Privacy
LDP: Local Differential Privacy
HE: Homomorphic Encryption
ZKP: Zero-Knowledge Proof
ABAC: Attribute-Based Access Control
PoS: Proof-of-Stake
GDPR: General Data Protection Regulation
PCI-DSS: Payment Card Industry Data Security Standard
